# Targeting Fungal Genes by Diced siRNAs: A Rapid Tool to Decipher Gene Function in *Aspergillus nidulans*

**DOI:** 10.1371/journal.pone.0075443

**Published:** 2013-10-10

**Authors:** Natarajaswamy Kalleda, Aruna Naorem, Rajam V. Manchikatla

**Affiliations:** Department of Genetics, University of Delhi South Campus, New Delhi, India; University of Georgia, United States of America

## Abstract

**Background:**

Gene silencing triggered by chemically synthesized small interfering RNAs (siRNAs) has become a powerful tool for deciphering gene function in many eukaryotes. However, prediction and validation of a single siRNA duplex specific to a target gene is often ineffective. RNA interference (RNAi) with synthetic siRNA suffers from lower silencing efficacy, off-target effects and is cost-intensive, especially for functional genomic studies. With the explosion of fungal genomic information, there is an increasing need to analyze gene function in a rapid manner. Therefore, studies were performed in order to investigate the efficacy of gene silencing induced by RNase III-diced-siRNAs (d-siRNA) in model filamentous fungus, *Aspergillus nidulans*.

**Methodology/Principal Findings:**

Stable expression of heterologous reporter gene in *A. nidulans* eases the examination of a new RNAi-induction route. Hence, we have optimized *Agrobacterium tumefaciens*-mediated transformation (AMT) of *A. nidulans* for stable expression of s*GFP* gene. This study demonstrates that the reporter *GFP* gene stably introduced into *A. nidulans* can be effectively silenced by treatment of *GFP*-d-siRNAs. We have shown the down-regulation of two endogenous genes, An*rasA* and An*rasB* of *A. nidulans* by d-siRNAs. We have also elucidated the function of an uncharacterized *Ras* homolog, *rasB* gene, which was found to be involved in hyphal growth and development. Further, silencing potency of d-siRNA was higher as compared to synthetic siRNA duplex, targeting An*rasA*. Silencing was shown to be sequence-specific, since expression profiles of other closely related *Ras* family genes in d-siRNA treated An*rasA* and An*rasB* silenced lines exhibited no change in gene expression.

**Conclusions/Significance:**

We have developed and applied a fast, specific and efficient gene silencing approach for elucidating gene function in *A. nidulans* using d-siRNAs. We have also optimized an efficient AMT in *A. nidulans*, which is useful for stable integration of transgenes.

## Introduction

RNA interference (RNAi) is a sequence-specific post-transcriptional gene silencing mechanism mediated by small interfering RNAs (siRNAs) which are the products of a double-stranded RNA (dsRNA) by the action of RNase III type enzyme Dicer. These siRNAs will then be incorporated into the RNA-induced silencing complex (RISC) and reduces cognate mRNA levels [1], [2]. In certain organisms, including fungi, the silencing phenomenon requires the action of RNA-dependent RNA polymerases (RdRPs), either to generate dsRNA from single-stranded RNA (ssRNA) or to amplify siRNA signals [3]. The specific silencing effect of RNAi on target genes makes it a powerful tool in functional genomic studies, drug target discovery and disease treatment [4], [5], [6], [7], [8]. The use of RNAi in mammalian cells for specific knockdown of target genes was limited before the discovery of synthetic siRNA-mediated gene silencing, as the introduction of long dsRNAs into the mammalian cells frequently triggers a lethal interferon response [9]. However, it has been found that siRNAs can specifically silence target genes without triggering interferon response [10], hence it becomes a promising approach for achieving RNAi in mammalian cells. The RNAi phenomenon in fungi “Quelling” was first demonstrated in ascomycete *Neurospora crassa* [11]. Targeting fungal genes by RNAi has been shown to be suitable for a multitude of fungal species [12].

The filamentous fungus *A. nidulans* is an important experimental model for studying eukaryotic cell biology [13]. It also serves as an important model organism for the genus *Aspergillus*, which includes industrially important fermentation organisms as well as serious human fungal pathogens. In addition, *A. nidulans* is an opportunistic human pathogenic fungus, which causes fatal invasive aspergillosis in patients suffering from chronic granulomatous disease (CGD) [14]. Moreover, *A. nidulans* has been a fungal model for RNAi using hairpin RNAi constructs [15], [16] and chemically synthesized siRNAs as silencing inducers [17].

The technical advances in sequencing platforms have rapidly brought in the discovery of a vast number of fungal genes without any predicted functions. Sequencing projects of most of the pathogenic and industrially important fungal species have now been completed (http://www.broad.mit.edu/annotation/fungi/fgi/). The next major challenge in understanding fungal biology is to translate this surplus genome sequence information into biological function information. Traditionally, the analysis of gene function requires the construction of mutant strains by disrupting or deleting the gene of interest. However, gene targeting in filamentous fungi is hampered by low frequencies of homologous recombination (HR) [18]. Recently, hairpin RNAi vectors and other plasmid-based silencing vectors have been exploited for studying gene function in multitude of filamentous fungi [19]. The current techniques available for construction of either conventional deletion cassettes or plasmid-based RNAi vectors are tedious and time consuming. Moreover, most of the fungi are recalcitrant for transformation.

Other than the plasmid-based RNAi vectors, the heterologous *GFP* reporter and endogenous genes coding for hydrophobins and a peroxiredoxin were silenced in *Moniliophthora perniciosa* transfected with *in vitro* transcribed respective dsRNAs. However, this method also requires protoplast preparations for dsRNA transfection [20]. Previously, it has been shown that the addition of chemically synthesized siRNA, targeting a key enzyme in polyamine biosynthetic pathway, to the culture medium, can result in specific suppression of the corresponding target gene with phenotypic consequences in *A. nidulans*. This method of small RNA uptake by fungal cells obviates the preparation of protoplasts for small RNA transfection [17].

Although chemically synthesized siRNAs are widely used in RNAi experiments, the *in silico* prediction and validation of siRNAs for a large number of RNAi targets is time consuming and laborious. Several guidelines and computational tools for designing effective siRNAs are available, but experiments reveal that a majority of the synthetic siRNAs designed against a target gene are not very satisfactory in terms of silencing potency and off-target effects [21], [22]. Many synthetic siRNAs do not work at low concentrations and are sometimes used at 100 nM or even higher concentrations [21], [23]. Synthetic siRNA that transfects at higher concentrations might aggravate the problem of off-target effects [24], [25], [26], [27]. However, siRNAs can also be made from *in vitro* transcribed dsRNAs by RNase III family enzymes [28], [29]. In this case, the resulting short RNA fragments contain several siRNAs against one target gene. A pool of d-siRNAs can sometimes be more effective and have fewer off-target effects than any single sequence synthetic siRNA [30], [31]. However, thus far, the possibility of d-siRNA mediated targeting of fungal genes has not been investigated.

To determine whether the d-siRNA induced RNAi route is functional in *A. nidulans* and could be efficiently used in targeting specific genes, the fungal mycelia were treated with d-siRNAs corresponding to a heterologous *GFP* reporter and the endogenous target genes (An*rasA* and An*rasB*). The reduction of GFP fluorescence and mRNA abundance of the target genes were then assessed. The results showed that this approach is simple and efficient in specifically silencing the target genes in *A. nidulans.* The silencing potency of d-siRNA was found to be greater than the equal concentration of single sequence synthetic siRNA duplex targeting the corresponding gene. In addition, we have optimized the efficiency of *Agrobacterium tumefaciens*-mediated transformation of *A. nidulans* for stable integration of transgenes.

## Results

### Construction of pCAMBIA1300-s*GFP* over-expression vector

**Figure 1:**
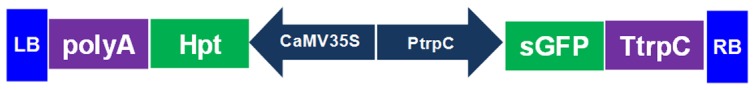
T-DNA map of s*GFP* expression vector. s*GFP* gene was cloned under the control of *Aspergillus nidulans* TrpC promoter and marker gene (*HPT*) was fused to CaMV35S promoter in the T-DNA back bone of pCAMBIA1300 binary vector.

The *GFP* over-expression vector was prepared by cloning an improved version of *GFP* gene, s*GFP*-TYG [32] in pCAMBIA1300 binary vector under the control of *A. nidulans* TrpC promoter. In s*GFP*-TYG, a Ser 65 to Thr point mutation was introduced in the chromophore domain which resulted in brighter fluorescence with a single excitation and emission peak [33]. pCAMBIA1300-s*GFP* (Figure 1) contains hygromycin B phosphotransferase gene (*HPT*) fused to CaMV35S promoter. To the best of our knowledge, CaMV35S viral promoter is being used for the first time in *A. nidulans* to drive the marker gene expression. Although, CaMV35S promoter is widely used in multitude of plant species for constitutive expression of transgenes, we observed minimal resistance to hygromycin B (100 µg/ml concentration) antibiotic in fungal transformants (fungal transformants resist upto 200 µg/ml hygromycin B concentration when *HPT* gene is fused to fungal-specific promoter). We found that 100 µg/ml hygromycin B is the optimal concentration for selection of fungal transformants (data not shown). We have also compared the *HPT* gene expression under the control of CaMV35S promoter with non-promoter expression in *A. nidulans*. We found that *HPT* gene expression without CaMV35S promoter was not sufficient to generate hygromycin B resistance (when selection medium was amended with 100 µg/ml hygromycin B) for *A. nidulans* transformants to undergo selection process.

### *Agrobacterium tumefaciens*-mediated transformation (AMT) of *A. nidulans*

To evaluate the possibility of d-siRNA mediated gene silencing in *A. nidulans*, we used the reporter gene *GFP* as a target for silencing. Therefore, we have generated GFP over-expressing *A. nidulans* transformants through AMT. Since AMT is not reported in *A. nidulans,* we have optimized the conditions for efficient transformation based on previous protocols reported in closely related filamentous fungus *A. fumigatus* [34]. The *GFP* over-expression vector pCAMBIA1300-s*GFP* was mobilized into *A. tumefaciens* EHA105 strain and the presence of vector was confirmed by colony PCR using *HPT* marker gene and *GFP* specific primers (data not shown). Fungal conidia and bacteria (1∶10) were co-cultivated for 48 h at 24°C on nylon membrane placed on induction medium amended with 100 mM acetosyringone. The nylon membranes were then transferred to selection medium containing 100 mg/l hygromycin B and incubated for another 3 days. Clear isolated fungal colonies appeared on nylon membranes after 3–4 days of incubation (Fig. S1A). Putative fungal transformants were sub-cultured for further growth (Fig. S1B) on selection medium and subjected to molecular analysis. We also transformed *A. nidulans* with pCAMBIA1300 vector containing *HPT* marker gene (not fused to any promoter) through AMT. However, we observed that no fungal transformants were appeared on nylon membranes placed on selection medium containing 100 mg/l hygromycin B and incubated for 4 days (Fig. S1C).

### Molecular analysis of fungal transformants and detection of GFP fluorescence

**Figure 2:**
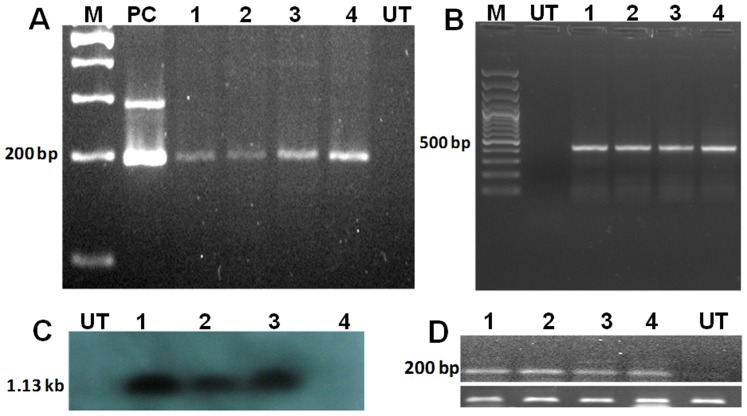
Molecular characterization of *A. nidulans* s*GFP* transformants. **(A)** DNA from several independent hygromycin resistant *A. nidulans* mycelia was extracted and the presence of s*GFP* gene (200 bp amplicon) was evaluated by PCR amplification. M. Ladder; PC. pCAMBIA 1300-s*GFP* plasmid control; 1–4. *A. nidulans* transformants represented as An*GFP* 101, An*GFP* 102, An*GFP* 103, An*GFP* 104; UT. Untransformed control. **(B)** DNA from several independent hygromycin resistant *A. nidulans* mycelia was extracted and the presence of *HPT* gene was evaluated by PCR amplification. M. Ladder; UT. Untransformed control; 1–4. An*GFP* 101, An*GFP* 102, An*GFP* 103, An*GFP* 104 fungal transformants. **(C)** Southern hybridization was performed using genomic DNA extracted from fungal transformants, DNA was (10 µg) digested with *Xho*I and probed with radio labelled *HPT* probe. UT. Untransformed control; 1–4. An*GFP* 101, An*GFP* 102, An*GFP* 103, An*GFP* 104 fungal transformants. **(D)** Semi-quantitative RT-PCR analysis for evaluation of expression of s*GFP* in *A. nidulans* transformants. Upper panel indicates s*GFP* expression, 1–4. An*GFP* 101, An*GFP* 102, An*GFP* 103, An*GFP* 104 fungal transformants; UT. Untransformed control. Lower panel: normalization of gene expression by An*Actin* as internal control.

**Figure 3:**
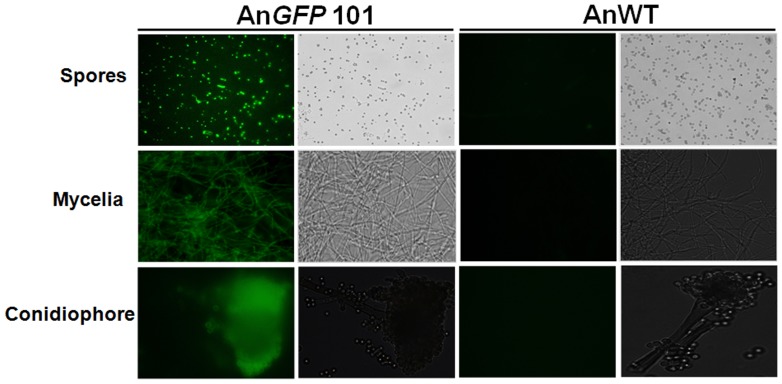
Detection of GFP in different developmental stages of *A. nidulans* (An*GFP* 101) transformant. GFP expression was examined using an OLYMPUS BX-51 fluorescent microscope under UV light (right) or white light conditions (left) in fungal spores, mycelia and conidiophores. Expression of GFP in An*GFP* 101 transformant (left panel) showed strong GFP fluorescence in all the developmental stages, whereas GFP expression was completely absent in untransformed control AnWT (right panel). All the comparative pictures in An*GFP* 101 and AnWT were taken at same magnifications using fluorescent microscopy.

To evaluate the presence and expression of transgenes in putative fungal transformants, four randomly-selected hygromycin B resistant fungal colonies were subjected to PCR and semi-quantitative RT-PCR analyses. PCR amplification with *HPT* and *GFP* specific primers (Table S1) was carried out on the total genomic DNA template extracted from putative fungal transformants which yielded the amplicons of expected sizes (Fig. 2A and 2B). Further, confirmation of transgene integration was demonstrated by Southern hybridization in several *A. nidulans* transformants (Fig. 2C). The transgene expression in putative fungal transformants was confirmed by RT-PCR analysis using *GFP* specific primers (Fig. 2D). Fluorescence microscopy was carried out in order to detect green fluorescence in fungal transformants and one of the strong GFP over-expressing fungal transformant (*AnGFP* 101) was chosen for further experiments. Green fluorescence was detected in various developmental stages of An*GFP* 101 *A. nidulans* transformant, such as in spores, mycelia and conidiophores (Fig. 3).

### *In silico* prediction of siRNA candidates and preparation of diced siRNAs

In order to achieve the efficient gene silencing, target gene sequences were analyzed for the presence of candidate siRNAs using siRNA prediction software [35]. Several candidate siRNAs were identified in coding sequences of *GFP* reporter, An*rasA* and An*rasB* genes. To further scrutinize the silencing potency of probable siRNA candidates of all the target genes, *in silico* siRNA scoring was performed according to the parameters suggested by Reynolds *et al.* [36]. *In silico* siRNA scoring revealed that some of the potent siRNA candidates in all of the three target genes possess the score above 7 which is hypothetically sufficient for effective gene knockdown (Table S2A, S2B and S2C).

**Figure 4:**
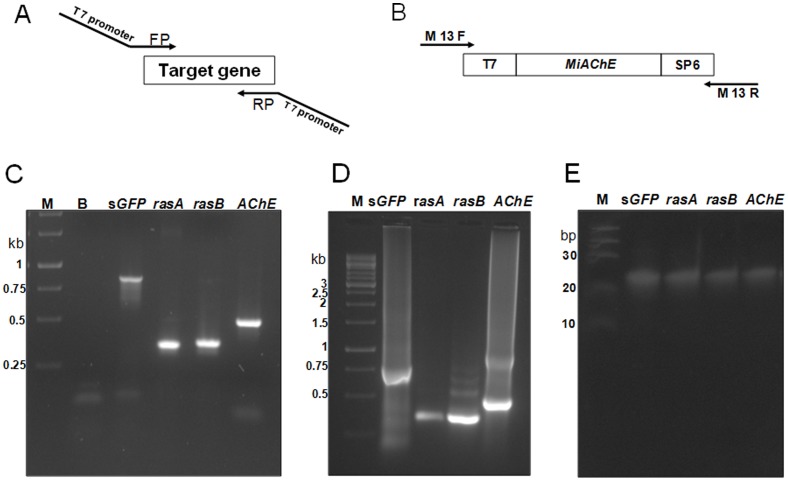
Strategies for generation of dsRNAs and d-siRNAs. **(A)** PCR template strategy was utilized for obtaining s*GFP*, An*rasA* and An*ras*B dsRNA in a single T7 transcription reaction. T7 promoter sequence was added to both forward and reverse gene specific primers of s*GFP*, An*rasA* and An*rasB*, and then PCR amplification was performed in order to generate templates for dsRNA synthesis. **(B)** For generation of template DNA for unrelated *MiAchE*, PCR amplification was performed on pGEM T-*MiAchE* vector using M13 primers. This amplification includes the T7 promoter to one end and SP6 promoter to another end of *MiAchE*. **(C)** PCR templates for transcription of respective target genes. M. ladder; B. blank; s*GFP*, An*rasA*, An*rasB* and *MiAchE* target gene templates. **(D)** Synthesis of dsRNAs with T7 or SP6 *in vitro* transcription reactions. M. Ladder; s*GFP*, An*rasA*, An*rasB* and *MiAchE* dsRNAs. **(E)** 20% PAGE analysis of purified diced siRNAs. M-Ladder, s*GFP*, An*rasA*, An*rasB* and *MiAchE* d-siRNAs. The d-siRNAs of all target genes were generated by cleaving respective dsRNAs with RNase III at 37°C for 30 mins, followed by subsequent purifications and finally dissolved in nuclease free water.

All the target genes were PCR amplified with gene-specific primers and cloned in pGEM-T easy TA cloning vector and confirmed by automated DNA sequencing. PCR amplification strategy was utilized for the generation of templates for dsRNA preparation for all the three target genes (Fig. 4A). T7 promoter sequence was added to both the forward and reverse gene specific primers and target genes were re-amplified from appropriate pGEM-T plasmids (Fig. 4C). *In vitro* transcription of all the three target genes were carried out using T7 RNA polymerase which recognizes the T7 promoter present at both the ends of target gene sequences and transcribes both the strands (partial dsRNAs forms due to complimentary base pairing). The intact dsRNAs were then produced by annealing the sense and antisense RNAs. Whereas for the preparation of unrelated dsRNA, *Meloidogyne incognita* acetylcholinesterase *(MiAchE)* gene cloned in pGEM-T easy plasmid (available in the lab) was used. *MiAchE* was PCR amplified using M13 primers from pGEM-T-*MiAchE* plasmid; this amplification adds the T7 promoter to one end and SP6 promoter to the other end of the *MiAchE* template (Fig. 4B). *In vitro* transcription of *MiAchE* was carried out using T7 RNA polymerase and SP6 RNA polymerase in separate reactions which transcribes sense and antisense RNAs respectively. *MiAchE* dsRNA was then produced by annealing the sense and antisense RNAs.

All the dsRNAs were treated with RNase-free DNase I in order to digest the DNA template in transcription reactions. The integrity of all the dsRNAs was confirmed through electrophoresis in 1.2 % agarose gel (Fig. 4D) and quantified by spectrophotometer. All the dsRNAs were digested with RNase III in separate reactions to prepare diced siRNA pools. The integrity was checked on 20% native PAGE (Fig. 4E) and quantified by spectrophotometer.

### Treatment of fungal mycelia with *GFP*-d-siRNA resulted in reduction of GFP fluorescence and *GFP* mRNA abundance

**Figure 5:**
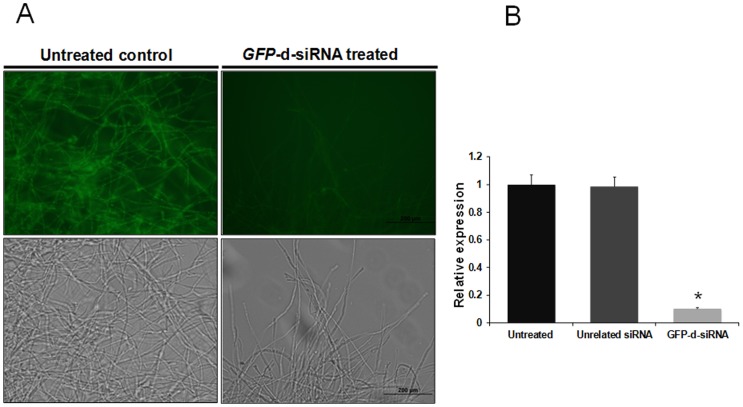
Evaluation of GFP silencing by *GFP*-d-siRNAs in *A. nidulans*. **(A)** Evaluation of green fluorescence in *GFP*-d-siRNA treated mycelia. *A. nidulans* (1×10^−4^/ ml) spores were added to 24 well culture plate and germination was induced at 37°C for 6 h, subsequently 25 nM final concentration of *GFP*-d-siRNAs were added and treated for another 12 h. Pictures by microscopy were taken 18 h after seeding. The (left panel) untreated fungal mycelia was showing brighter GFP fluorescence, whereas (right panel) the treatment of fungal mycelia with 25 nM final concentration *GFP*-d-siRNA resulted in drastic reduction in GFP fluorescence. Upper panel showing the microscopic pictures under the UV light and lower panel showing the microscopic pictures under the white light. **(B)** Quantification of GFP silencing by qRT-PCR. Relative GFP expression was measured in untreated control, unrelated siRNA treated and *GFP*-d-siRNA treated mycelia using comparative D cycle threshold (CT) method. GFP values were normalized to An*Actin* values. The data represent the means of three replicates. Values were compared using *t* test. * Significant difference at *P<0.05* as compared to untreated control.

To demonstrate the possibility of d-siRNA-mediated gene silencing, we first targeted the heterologous *GFP* reporter gene in *A. nidulans* mycelia. We found that 25 nM final concentration of d-siRNA treatment was optimum for achieving efficient silencing through the direct siRNA uptake transfection method (data not shown) and the same concentration was utilized throughout the study. In order to observe the *GFP* reduction in *GFP*-d-siRNA treated samples, fluorescent microscopy analysis was performed. The *GFP*-d-siRNA treated mycelia showed strong reduction in GFP fluorescence whereas untreated mycelia showed the normal levels of fluorescence (Fig. 5A).

To ensure that the silencing occurred at the transcript level, the expression of *GFP* RNA was measured by quantitative RT-PCR (qRT-PCR) in *GFP*-d-siRNA treated, unrelated *MiAchE*-d-siRNA treated and untreated mycelia. The *GFP* mRNA abundance was found to be reduced by 89.60% in *GFP*-d-siRNA treated mycelia when compared to unrelated *MiAchE*-d-siRNA treated and untreated control mycelia (Fig. 5B).

### d-siRNA-mediated silencing of the endogenous An*rasA* and An*rasB* genes hampered *A. nidulans* growth and development

**Figure 6:**
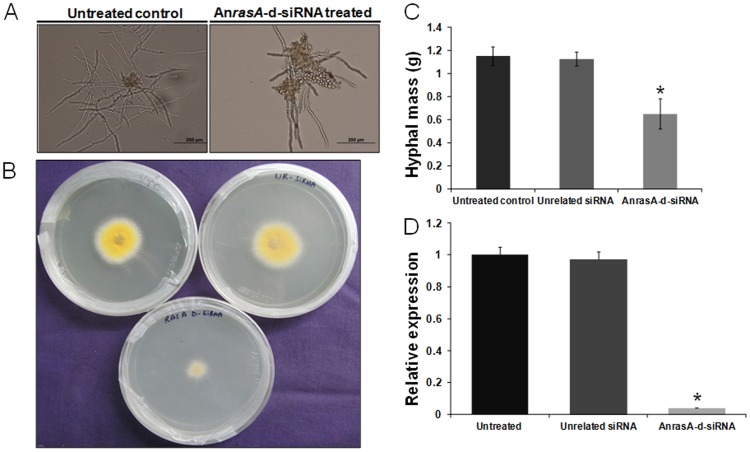
Evaluation of An*rasA* silencing by An*rasA*-d-siRNA in *A. nidulans*. **(A)** Determination of phenotypic changes in *A. nidulans*. The germinating conidia were treated with An*rasA*-d-siRNA for 12 h, then light microscopic analysis (18 h after seeding) revealed that the majority of treated conidia were swollen but unable to form hyphal tissue compared to control. **(B)** Radial growth assay. Germinating conidia were treated with An*rasA*-d-siRNA and spotted onto center of the ACM agar plate and radial outgrowth was monitored 48 h post-inoculation. Radial growth was drastically reduced in An*rasA* silenced lines compared to controls. **(C)** Comparison of the total biomass. Germinating conidia were treated with An*rasA*-d-siRNA in ACM broth for 48 h and total fresh biomass formation was determined. The data represent the means of three replicates. Values were compared using *t* test. * Significant difference at *P<0.05* as compared to untreated control. **(D)** Quantification of An*rasA* silencing by qRT-PCR. Relative An*rasA* expression was measured in untreated control, unrelated siRNA treated and An*rasA*-d-siRNA treated mycelia using comparative D cycle threshold (CT) method. An*rasA* values were normalized to An*Actin* values. The data represent the means of three replicates. Values were compared using *t* test. * Significant difference at *P<0.05* as compared to untreated control.

To assess the possibility of silencing the endogenous genes by d-siRNAs in *A. nidulans,* we have chosen the Ras protein coding An*rasA* and An*rasB* genes, which are involved in conidial germination and asexual development, as targets. An*rasA* deletion is lethal to *A. nidulans,* so it is impractical to isolate deletion strain. However, it has been shown that mutations in An*rasA* gene cause aberrations in germination and asexual development in *A. nidulans* [37]. The regulated expression of *rasA* also plays a pivotal role in conidial germination, which is required to initiate both infection and asexual development in the opportunistic pathogen *A. fumigatus* [38]. In order to study crucial gene (where generation of deletion mutant is not possible) function in filamentous fungi, siRNA/d-siRNA mediated knockdown is a suitable method since it only transiently suppresses the target gene activity. The treatment of *AnrasA*-d-siRNA to the germinating spores resulted in reduction in hyphal elongation and mycelial formation in broth culture (Fig. 6A). We also observed significant reduction in radial growth in *AnrasA*-d-siRNA treated culture 48 h post-inoculation on ACM agar medium compared to controls (Fig. 6B). In addition, total hyphal mass was also significantly reduced by 43.80% in An*rasA-*d-siRNA silenced lines as compared to controls after 48 h post-inoculation in ACM broth (Fig. 6C).

**Figure 7:**
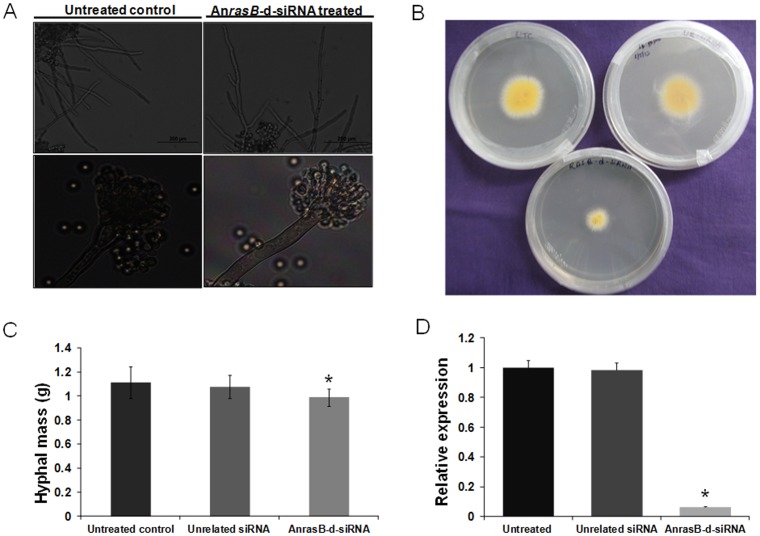
Evaluation of An*rasB* down-regulation by An*rasB*-d-siRNA in *A. nidulans*. **(A)** Assessment of phenotypic changes in *A. nidulans*. In upper panel germinating conidia were treated with An*rasB*-d-siRNA for 12 h, and then light microscopic analysis was performed. The An*rasB* silenced lines possess the irregular apical branching when compared to control. In the lower panel, fungal tissue was collected from ACM solid agar media containing An*rasB* silenced lines and control lines, then viewed using OLYMPUS BX-51 microscope under the white light conditions. **(B)** Radial mycelial growth assay. Germinating conidia were treated with An*rasB*-d-siRNA and spotted onto center of the ACM agar plate and radial outgrowth of mycelia was monitored 48 h post-inoculation. Radial growth was significantly reduced in An*rasB* silenced lines compared to controls. **(C)** Determination of the total biomass. Germinating conidia were treated with An*rasB*-d-siRNA in ACM broth for 48 h and total fresh biomass formation was determined. The data represent the means of three replicates. Values were compared using *t* test. * Significant difference at *P<0.05* as compared to untreated control. **(D)** Quantification of An*rasB* silencing by qRT-PCR. Relative An*rasB* expression was measured in An*rasB*-d-siRNA treated mycelia and controls using comparative D cycle threshold (CT) method. An*rasB* values were normalized to An*Actin* values. The data represent the means of three replicates. Values were compared using *t* test. * Significant difference at *P<0.05* as compared to untreated control.

Another fungal specific *Ras* family homolog *rasB* plays a pivotal role in germination, growth rate, regulated hyphal morphology and virulence in *A. fumigatus* [39]. *A. nidulans* complete genome sequence and annotation revealed the presence of *rasB* homolog in the genome, and it was predicted to have a 723-base pair coding sequence. To date, there is no report on the characterization of *rasB* in *A. nidulans.* Therefore, we have chosen this *rasB* homolog as the second endogenous target for d-siRNA-mediated silencing. The knockdown of An*rasB* resulted in abnormal hyphal morphology characterized by irregular apical branching. The An*rasB-*d-siRNA treatment also resulted in malformed conidiophores (Fig. 7A). In addition, we have analyzed the radial growth and total biomass of the isogenic An*rasB*-d-siRNA treated cultures grown for 48 h at 37°C. The radial growth assay performed on ACM solid agar medium revealed that An*rasB-*d-siRNA treatment significantly hampers the radial outgrowth in An*rasB* silenced lines as compared to controls (Fig. 7B). An*rasB-*d-siRNA treatment did not significantly affect the total hyphal mass accumulation of silenced cultures grown in ACM broth for 48 h at 37°C (Fig. 7C).

### An*rasA* and An*rasB* mRNA abundance decreases after treatment with d-siRNAs

To demonstrate the silencing of the endogenous An*rasA* and An*rasB* mRNAs, total RNA was isolated from An*rasA or* An*rasB*-d-siRNA treated, unrelated *MiAche*-d-siRNA treated and untreated control mycelia and cDNA was produced. The relative expression levels of target mRNAs were measured by qRT-PCR in An*rasA or* An*rasB*-d-siRNA silenced lines. The An*rasA* mRNA abundance was found to be reduced by 96.07% in An*rasA*-d-siRNA treated mycelia when compared to unrelated *MiAche*-d-siRNA treated and untreated control mycelia (Fig. 6D). Similarly, An*rasB* mRNA level was also reduced by 93.53% in comparison to controls (Fig. 7D).

### d-siRNA is more potent in silencing target mRNA than chemically synthesized siRNA counterpart

To determine the silencing potency of d-siRNA when compared to synthetic siRNA, we have chosen to target An*rasA* gene in *A. nidulans*. Target gene sequence was *in silico* scrutinized for high score siRNA in order to attain maximum silencing potency [36]. However, *in silico* predicted siRNA might have different outcome with *in vitro* assays, even if it possesses a high score. So, we eliminated the three non-efficient chemically synthesized siRNAs (data not shown) and we compared the silencing potency of the best working (*in vitro* assay) synthetic An*rasA*-siRNA versus its d-siRNA counterpart. *In silico* designed An*rasA*-siRNAs were chemically synthesized and dissolved in nuclease-free water. *A. nidulans* conidia were grown for 6 h in ACM and subsequently treated with synthetic An*rasA-*siRNA and diced An*rasA*-siRNA with final concentrations of 25 nM for another 12 h. Mycelial tissue was then collected from synthetic or diced An*rasA* siRNA treated samples and controls. Total RNA was isolated from frozen mycelial samples and cDNA was synthesized. To analyze the An*rasA* mRNA abundance in control and siRNA treated samples, qRT-PCR was performed.

**Figure 8:**
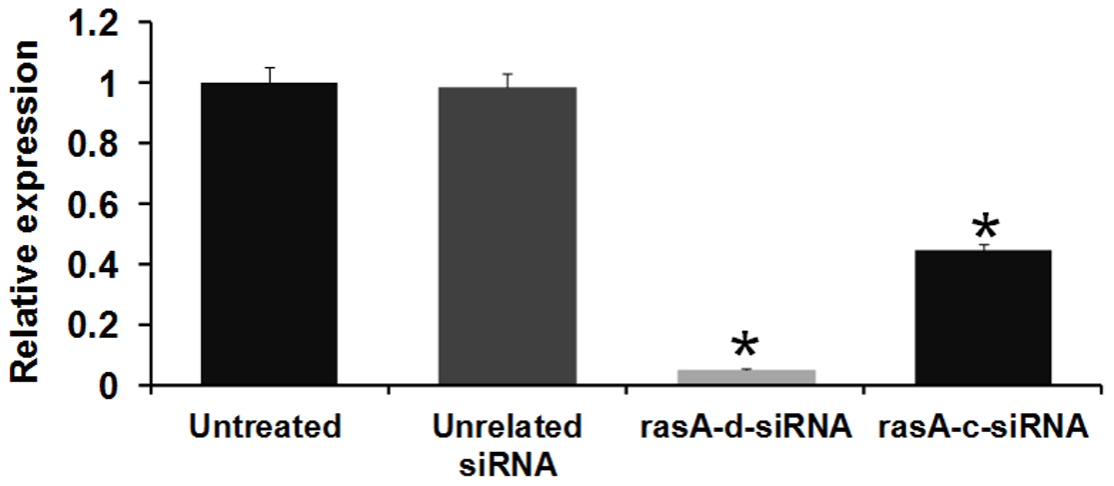
Determination of silencing efficacies of synthetic siRNA and d-siRNA. Real-Time PCR analysis was performed to compare the silencing potency of chemically synthesized siRNA and RNase III-diced-siRNA targeting *rasA* endogenous gene in *A. nidulans*. *A. nidulans* spores were germinated for 6 h in ACM medium. Subsequently, 25 nM final concentration of synthetic An*rasA* siRNA (*rasA-*c-siRNA) and RNase III generated An*rasA* siRNA (*rasA*-d-siRNA) were added and incubated for another 12 h. Then mycelial tissues were collected and RNA was isolated. Relative An*rasA* expression was measured in untreated control, unrelated siRNA treated, *rasA*-d-siRNA treated and *rasA*-c-siRNA treated mycelia using comparative D cycle threshold (CT) method. An*rasA* values were normalized to An*Actin* values. The data represent the means of three replicates. Values were compared using *t* test. * Significant difference at *P<0.05* as compared to untreated control.

qRT-PCR results showed that the level of An*rasA* transcript was reduced to 5.30% in An*rasA*-d-siRNA silenced lines compared to controls. However, we found that the same concentration of single synthetic An*rasA*-siRNA treatment reduced An*rasA* transcript levels only to 44.38% (Fig. 8). These results suggest that d-siRNAs *in vitro* generated by RNase III enzyme is more potent in silencing endogenous genes than single synthetic siRNA in *A. nidulans*.

### Expression profiles of other *Ras* family genes in An*rasA* and An*rasB* silenced lines exhibited no change in transcript levels

**Figure 9:**
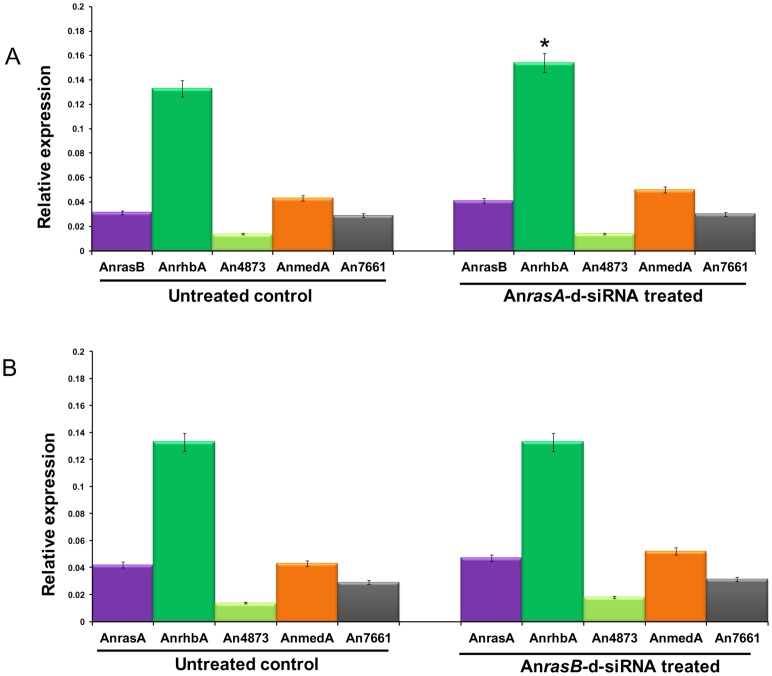
Expression profiles of *Ras* family genes in d-siRNA treated lines. **(A)** Estimation of relative expression levels of *Ras* family genes in untreated control and An*rasA*-d-siRNA treated *A. nidulans.* Relative quantification was performed by qRT-PCR using comparative D cycle threshold (CT) method. Expression levels of An*rasB*, An*rhB*, An*4873*, An*medA* and An*7661* in untreated control was showed in the left panel, right panel represents the expression levels of same genes in An*rasA*-d-siRNA treated *A. nidulans*. Expression levels of all the genes were normalized to An*Actin* levels. The data represent the means of three replicates. Values were compared using *t* test. * Significant difference at *P<0.05* as compared to untreated control. **(B)** Estimation of relative expression levels of *Ras* family genes in untreated control and An*rasB*-d-siRNA treated *A. nidulans*. Relative quantification was performed by qRT-PCR using comparative D cycle threshold (CT) method. Expression levels of An*rasA*, An*rhB*, An*4873*, An*medA* and An*7661* in untreated control was showed in the left panel, right panel represents the expression levels of same genes in An*rasB*-d-siRNA treated *A. nidulans*. Expression levels of all the genes were normalized to An*Actin* levels. The data represent the means of three replicates.

In order to check the specificity of d-siRNA-mediated gene silencing, we analyzed the expression profiles of closely related, non-intended targets of other *Ras* family genes in An*rasA* and An*rasB*-d-siRNA silenced lines. Expression profiles of An*rhbA*, *An4873*, An*medA* and *An7661* in both An*rasA* and An*rasB*-d-siRNA silenced lines, An*rasA* expression in An*rasB*-silenced line and vice-versa were obtained through qRT-PCR analysis. We showed that the transcript levels of all genes tested were found to be normal as in untreated control (Fig. 9A and 9B). However, transcript level of An*rhbA* in An*rasA*-d-siRNA silenced line showed slightly elevated level than in untreated control but no such elevation was observed in An*rasB* silenced lines. We also showed that the transcript levels of all *Ras* family genes tested in un-diced An*rasA* or An*rasB* dsRNA treated lines were found to be normal as in untreated control (Fig. S2A and S2B). These results suggest that d-siRNA mediated gene silencing seem to be target-specific, as no other similar or closely related genes were affected.

## Discussion

RNAi is an emerging and powerful reverse genetic approach to characterize gene functions in various filamentous fungi [12]. With the explosion of sequenced fungal genomes (www.broad.mit.edu/annotation/fungi/fgi/), there is an increasing interest in looking beyond the genome and investigating the function of genes. Conventionally, deciphering gene function in filamentous fungi has been performed by targeted gene disruption either by insertion or knockout method. This strategy is time-consuming and not feasible for large scale functional genomic studies. The majority of the genes present in the genome are critical for the survival of the fungus and deleting any gene for functional study may result in a lethal phenotype. Hence, an alternative method which can generate down-regulation of gene activity rather than complete deletion of target gene might be more informative [40]. Thus, siRNA-induced gene silencing offers a convenient method for targeting specific genes in filamentous fungi. Previous reports have shown that siRNA-induced gene silencing targeted against ornithine decarboxylase (*ODC*) gene, a key polyamine biosynthesis gene in *A. nidulans*. This revealed the importance of cellular polyamines for proper growth of germ tube and mycelia. It also demonstrated successful uptake of siRNA molecules (25 nM optimum concentration) by germinating fungal spores from culture medium, which ultimately supports the simple transfection of small RNA species into the germinating spores or mycelia [17]. Subsequently, another group also reported that this small RNA uptake mechanism can be exploited to induce siRNA-mediated silencing of endogenous genes in *A. fumigatus* [41]. In our first attempt, we employed this simple small RNA uptake mechanism for silencing of fungal genes by RNase III generated diced siRNAs in *A. nidulans*.

The evaluation of a new RNAi induction route is made more comfortable by silencing of a stably introduced reporter gene, which can facilitate the visible quantification of silencing effect. Hence, we optimized stable introduction of *GFP* reporter gene into *A. nidulans* genome by AMT. Although it has been previously demonstrated that *Agrobacterium tumefaciens* is capable of transforming various fungi including the *Aspergillus* species [34], [42], the transformation conditions must be optimized because the transformation frequencies vary among fungal species and strains. We have prepared a transformation vector (pCAMBIA1300-sGFP) which contains the *HPT* as a marker gene driven by CaMV35S promoter and *sGFP* gene driven by fungal-specific TrpC promoter using pCAMBIA 1300 T- DNA backbone. The CaMV35S promoter is widely used in multitude of plant species for driving constitutive expression of transgenes. However, only one such report is available in phytopathogenic fungus *Fusarium oxysporum* where they have shown that CaMV35S promoter efficiently drives the marker gene expression [43]. Hence, we utilized the CaMV35S promoter to drive the *HPT* marker gene expression in *A. nidulans*. In consistent with the earlier results, *A*. *nidulans* transformants showed small colony size, indicating the CaMV35S promoter is not as efficient in driving selection marker gene expression as in plants. However, we showed that non-promoter expression of *HPT* marker gene (leaky expression of *HPT* gene) was not sufficient to generate *A. nidulans* transformants on selection medium containing 100 mg/l hygromycin B. This observation suggests that the CaMV35S promoter may be useful in driving the marker gene expression in *A. nidulans* as the small colony size of the transformants is not interfering with the study. Moreover, most of the *A. tumefaciens* binary vectors contain CaMV35S promoter for driving (hygromycin B) marker gene expression, the vectors can directly be used in transformation of *A. nidulans* without any further modifications.

We have successfully optimized the AMT in *A. nidulans* based on previous protocol described in *A*. *fumigatus* [34] with the transformation frequency of approximately 80 transformants for 10^7^ conidia. The frequency of transformation was comparable to that of the transformation frequency attained in *A. fumigatus* in the previous study. Molecular characterization by PCR and RT-PCR analyses confirmed the presence and expression of transgene in some of the putative fungal transformants. Further, transgene integration in the transformants was confirmed by Southern hybridization. *A. nidulans* transformant under study was found to have stable expression of GFP in different developmental stages tested.

The silencing potency of siRNA is mainly dependent on how effectively the siRNA is incorporated into RISC [44], [45], [46]. Besides the available computational siRNA prediction tools, it is necessary to test the several synthetic siRNAs in order to identify a potent siRNA duplex. Synthetic siRNAs can produce substantial off-target effects as a result of cross hybridization between the seed region of the siRNA and the 3′UTR of the off-target transcripts [28], [47]. Recently, it has been shown that testing of multiple siRNAs and off-target effects can be circumvented by generation of siRNAs using RNase III or Dicer enzyme [28]. The digestion of *in vitro* transcribed dsRNA with RNase III produces a mixture of siRNAs covering a much larger region of the target sequence. This usually obviates the need to test multiple sequences for each target gene, making the silencing more effective and having fewer off-target effects than any one single synthetic siRNA [30], [31].

We provided evidences for an efficient gene silencing induced by RNase III generated siRNAs in *A. nidulans*. In the first step, we showed that the *GFP* reporter gene stably introduced into *A. nidulans* genome was silenced by treating with *GFP-*d-siRNAs. GFP fluorescence in mycelia treated with *GFP-*d-siRNA was drastically reduced when compared to untreated mycelia, indicating that the silencing by RNase III-diced-siRNA is highly efficient. This was supported by reduction in mRNA level of *GFP* (89.6%) in silenced lines in comparison to controls. Our results are in agreement with the previously reported *GFP* dsRNA or hairpin *GFP* construct induced silencing in multitude of fungi, such as *Moniliophthora perniciosa* [20], *Colletotrichum lagenarium* [48], *Magnaporthe oryzae* [40], *Venturia inaequalis* [49], *Phytophthora infestans* [50] and *Coprinopsis cinerea* [51].

To be useful as a rapid reverse genetic tool, d-siRNA-mediated gene silencing must work for endogenous genes. Towards this goal, we attempted to silence an endogenous *Ras* homolog An*rasA* gene in *A. nidulans*. We reported that the An*rasA*-d-siRNA treatment resulted in severe reduction in hyphal growth, reduced radial outgrowth and total biomass accumulation. The phenotypic abnormalities exhibited by the An*rasA*-d-siRNA silenced mycelia were supported by strong reduction in corresponding mRNA abundance. To date, the isolation of a viable deletion mutant of *rasA* gene has not been reported in *A. nidulans*, suggesting that *rasA* activity is essential; thus, deletion would be lethal. Although *rasA* deletion mutant in *A. nidulans* has never been achieved, dominant-active or dominant-negative *rasA* mutant inhibits asexual development [37], [52]. Our results demonstrate that functional analysis of essential genes may be possible with their transient down-regulation using d-siRNAs.

To further assess the efficacy of d-siRNA-induced endogenous gene knockdown, we attempted the silencing of an uncharacterized *Ras* homolog, *rasB,* in *A. nidulans*. We found that down-regulation of An*rasB* by *rasB*-d-siRNA resulted in abnormal hyphal branching, malformed conidiophore and reduction in radial outgrowth, which was in consistent with the drastic reduction in mRNA levels (93.53% reduction) of *rasB* gene when compared to controls. Our results are in line with those obtained with *rasB* deletion mutant phenotype in *A. fumigatus*. The deletion of *rasB* in *A. fumigatus* has been reported to exhibit delayed growth, decrease in germination rate and abnormal hyphal morphology [39].

Silencing efficacy of siRNA is crucial for any successful RNAi experiment. In order to assess the silencing efficacy of the RNase III-diced-siRNA in *A. nidulans,* we treated the fungal mycelia with equal concentrations of synthetic siRNA or d-siRNA targeting an endogenous gene (An*rasA)* separately. We found that the silencing potency of An*rasA* by d-siRNA was relatively higher than that by synthetic siRNA. In mycelia treated with d-siRNA, An*rasA* mRNA level was reduced to 5.30%, whereas, the same concentration of synthetic siRNA treatment resulted in reduction of mRNA level to only 44.38%. These results are in agreement with previous results obtained with RNase III-diced-siRNAs in cultured mammalian cells [29].

RNAi mainly relies on homology-dependent base pairing leading to cognate mRNA degradation and it may sometimes cause non-intended silencing effects as well. In order to investigate off-target effects caused by d-siRNA, we evaluated its effect on other closely related *Ras* gene family in An*rasA* or An*rasB* gene silenced lines. We found that silencing of An*rasA* or An*rasB* gene with respective d-siRNAs did not alter the expressions of other *Ras* family genes. However, we observed slightly elevated expression of some of the *Ras* family genes tested. The probable explanation for this observation is due to the down-regulation of important gene which might lead to elevation in the expression levels of some genes through unknown mechanism. Previously, it has been reported that silencing of heterologous *GFP* gene with *GFP*-dsRNA resulted in insignificant elevation of *inf1* endogenous gene in *P. infestans* [50]. Though, the phylogenic and sequence analyses (Fig. S3 and S4) revealed that An*rasA* and An*rasB* were close homologs; the silencing of An*rasA* did not alter the expression of An*rasB* transcript level and vice-versa. Similarly, we also found that the treatment of un-diced An*rasA* or An*rasB* dsRNA did not alter the expression profiles of other *Ras* family genes. These results are in line with dsRNA-induced sequence specific silencing in *P. infestans* [50] and tobacco rattle virus mediated transient silencing of *Manduca sexta* midgut-based CYP6B46 gene [53]. Altogether, our data suggests that the silencing induced by d-siRNA is highly specific and did not affect even closely related genes of the same family.

We conclude that silencing of *A. nidulans* genes by RNase III-diced-siRNA is highly specific, efficient and does not rely on laborious methods. This method has a great potential to be used as a tool for deciphering the function of fungal genes. This method also facilitates the analyzing of essential genes in *A. nidulans* through down-regulation of the intended target. We succeeded in elucidating the function of an uncharacterized *Ras* homolog *rasB* gene in *A. nidulans*. The off-target effects of d-siRNA mediated gene silencing are undetectable but can further be reduced by prior off-target analysis before generation of d-siRNA pools. Using this simple and effective gene silencing method, rapid characterization of a large number of candidate genes could be possible in filamentous fungi.

## Materials and Methods

### Fungal culture and spore isolation

*A. nidulans* strain (strain-b1 A1 ribo A1-wild yellow) was cultured on solid complete medium according to Barratt *et al.* [54] in 90×15 mm Petri dishes. For spore collection, sterile double-distilled water (8 ml) was added to Petri dish containing *A. nidulans* culture, and the surface was scraped gently with a sterile glass rod to release the spores. The resulting spore suspension was filtered through glass wool and centrifuged at 12000 rpm for 10 min at 4°C. The spores were washed with sterile water and finally re-suspended in sterile nuclease-free water. For liquid growth, *A. nidulans* was cultured at 37°C upto the indicated time-points in ACM broth.

### Development of pCAMBIA1300-s*GFP* construct

The pCAMBIA1300-s*GFP* expression vector was generated using the T-DNA vector pCAMBIA1300 as the skeleton. The s*GFP* gene was PCR amplified from the pMT-s*GFP* vector (procured from the Fungal Genetics and Stock Centre, USA) and cloned in pGEM T-easy vector (Promega, USA). Then, s*GFP* gene was sub-cloned in pSilent-1 vector in *Xho*I and *Kpn*I restriction sites by removing the spacer DNA and finally the expression cassette was introduced into pCAMBIA1300 backbone at the *Xba*I restriction site (Fig. S5), which possess *HPT* gene as the antibiotic selection marker. For the comparison of *HPT* marker gene expression under the control of CaMV35S promoter with non-promoter expression, the CaMV35S promoter fused to the *HPT* marker gene was released from the pCAMBIA1300 vector by digesting with *Sma*I restriction enzyme. Then the resulting linearized pCAMBIA1300 vector was self-circularized using T4 DNA ligase (Fermentas, USA).

### Transformation of *A. nidulans*

AMT was carried out using the basic protocol as previously described in *A. fumigatus* [34] with some modifications. All the media used in various steps of transformation were prepared as mentioned in the previous protocols [34]. *A. tumefaciens* EHA105 strain harboring pCAMBIA1300-s*GFP* vector was grown in YEM broth supplemented with 50 µg/ml of kanamycin and 100 µg/ml rifampicin on a rotatory shaker (250 rpm) at 28° C for 12 h. One ml of the *Agrobacterium* culture was inoculated into 9.0 ml of induction medium (IM) with appropriate concentration of acetosyringone (AS) and incubated on a shaker (250 rpm) at 28°C for 6 h or until an optical density at 660 nm of 0.8 was reached. Fungal conidia and *Agrobacterium* were co-cultivated at a ratio of 1∶10 respectively. A total of 100 µl of the *Agrobacterium* culture with OD = 0.8 was mixed with 100 µl of 10^7^ fungal conidia and spread on to the nylon membrane (MDI, India) placed on IM-AS agar plate. Then, the IM-AS co-cultured plates containing the nylon membrane were incubated at 24° C in the dark for 48 h. In order to select the fungal transformants, nylon membranes were transferred to selection medium (SM) [ACM+100 mg/l hygromycin B+300 mg/l augumentin] plates and incubated at 37°C for 3 days.

### DNA isolation, PCR and Southern hybridization

Several independent putative fungal colonies raised in AMT were maintained on SM agar plates with appropriate hygromycin B concentration. For genomic DNA isolation, untransformed wild-type strain and fungal transformants were inoculated on to the ACM and grown at 37°C for 48 h in a rotary shaker. Genomic DNA was isolated from untransformed control and four putative fungal transformants using fungal DNA isolation kit (Himedia Labs, India) according to the manufacture's instructions. The putative fungal transformants were screened for the presence of transgenes using *HPT* and *GFP* specific primers.

Transgene integration in several fungal transformants was further confirmed by Southern analysis. Genomic DNA (10 µg) was digested with *Xho*I restriction enzyme and separated on 0.8% agarose gel. Then, blots were prepared by standard protocol [55] using nylon membrane (MDI, India). The PCR amplified and purified 500 bp *HPT* DNA was radio labeled with radioactive α-p32-dCTP using Megaprime DNA labeling kit (G. E Healthcare, USA) according to the manufacturer's instructions. Pre-hybridization and hybridization were carried out as previously described [55].

### *In silico* analysis and cloning of An*rasA* and An*rasB* partial genes

The coding sequences of An*rasA* and An*rasB* were retrieved from *Aspergillus* genome database (www.aspgd.org/). The presence of candidate siRNAs was evaluated using an algorithm developed by Bingbing Yuan *et al*. [35]. Further, siRNA scoring was done as previously described [36].

Total RNA was isolated from *A. nidulans* sub-merged culture using fungal RNA isolation kit (Himedia, India) and cDNA was prepared using Superscript (Invitrogen, USA) according to manufacturer's instructions. The partial coding sequences of An*rasA* and An*rasB* were PCR amplified using respective gene-specific primers (Table S1) from *A. nidulans* cDNA. The An*rasA* and An*rasB* PCR products were purified using PCR clean up system (Fermentas, USA) and cloned in pGEM-T easy (Promega, USA) vector and confirmed by automated DNA sequencing.

### Double-standard RNA (dsRNA) preparation

The dsRNAs were synthesized via *in vitro* transcription with the highscribe T7 *in vitro* transcription system (Fermentas, USA) using PCR products as templates for s*GFP*, An*rasA* and An*rasB* target genes. The T7 promoter sequence was placed in front of both the gene specific forward and reverse primers which were used for PCR amplification of the template for dsRNA synthesis. *In vitro* transcription was carried out according to the manufacturer's instructions. For synthesis of unrelated dsRNA, *Meladogina incognata* acetylcholineesterase (*MiAchE*) was PCR amplified from *M. incognata* cDNA and cloned in pGEM-T easy vector (already available in the lab). *MiAchE* was re-amplified from pGEM T-*MiAChE* with M13 primers and PCR products were used separately in T7 and SP6 transcription systems in separate reactions. Annealing of single standard RNAs was carried out in TE buffer at 65°C followed by snap cooling. The dsRNA quality was monitored by agarose gel electrophoresis and the concentration was determined by spectrophotometry.

### Preparation of diced-siRNA pools and chemically synthesized siRNA

The dsRNAs were digested with Short Cut RNase III (New England Biolabs, USA) to prepare diced-siRNA pools according to the manufacturer's protocol. Briefly, 10 µg dsRNAs were digested with Short Cut RNase III in a 100 µl reaction at 37°C for 30 min. Then 10 µl of 10X EDTA (0.5 M) was added to stop the reaction, and the small RNAs were precipitated in presence of nuclease-free glycogen and one-tenth volume of 3 M Sodium acetate (pH 5.2) with 3 volumes of chilled ethanol. Finally purified d-siRNAs were dissolved in nuclease-free water and integrity was analyzed by 20% native PAGE.

The custom-designed and chemically synthesized An*rasA* gene-specific siRNA used in this study was procured from Sigma, USA. The stock solutions of 100 µM were prepared and stored at −80° C until use. The 5′-3′ sequences of the sense and antisense strands of An*rasA* are given below: Sense: 5′ CCAUGCGCGAACAAUAUAUTT 3′Antisense: 5′ AUAUAUUGUUCGCGCAUGGTT 3′

### Treatment of *A. nidulans* mycelia with siRNAs

*A. nidulans* mycelia was treated with synthetic siRNA or d-siRNAs by the procedure previously described [17], [41] with minor modifications. Briefly 1×10^8^ spores/ml (instead of 1×10^7^ spores/ml) were incubated at 37°C and 250 rpm in liquid ACM for 6 h to obtain germinating spores. Subsequently, siRNAs were added to the liquid medium at a final concentration of 25 nM. Germinating conidia were treated for another 12 h. Later, cultures were harvested and stored at −80°C until RNA extraction.

### qRT-PCR analysis

Total RNA was extracted from siRNA treated and untreated samples by using fungal RNA isolation kit (Himedia labs, India) according to the manufacturer's instructions. cDNA was generated from total RNA using Superscript reverse transcriptase (Invitrogen, USA) according to manufacturer's instructions.

qRT-PCR was performed using SYBR Green I technology in Realplex^2^ thermal cycler (Eppendorf, Germany). A master mix was prepared by using MESA Blue qPCR mix as a PCR core reagent (Eurogentec, USA). 1.5 ng of cDNA and 750 nM each of the specific primers were added in a final volume of 10 µl (Table S1). The amplification reactions were carried out at 95°C 4 min, 40 cycles at 95°C 15 sec followed by 60°C for 1 min. Specificity of amplification was assessed by disassociation or melt curve analysis at 60–95°C after 40 cycles. After completion of qRT-PCR, data was analyzed by using Realplex^2^ software. Relative quantification of mRNA levels was performed by the comparative D cycle threshold (CT) method using An*Actin* mRNA as an internal control.

### Microscopic analysis of d-siRNA treated fungal culture

*A*. *nidulans* spores (final concentration of 1×10^4^/ml per well) were incubated in 1 ml of ACM broth in a 24 well cell-culture plate and incubated at 37°C for 6 h in order to obtain germinating conidia. Subsequently, d-siRNAs directed against s*GFP* or An*rasA* or An*rasB* transcripts were added to the liquid medium to get a final concentration of 25 nM. Fungal cultures were treated for another 12 h. Pictures by microscopy were taken 18 h after seeding.

### Radial outgrowth and total biomass accumulation assays

Radial outgrowth assay was performed as previously described [17] with some modifications. Briefly, 5 µl of 10^4^ germinating spores ml^−1^ (instead of 3 µl of 10^8^ germinating spores ml^−1^) was treated with respective d-siRNAs and were spotted in the center of ACM agar plates. The change in colony diameter was monitored after 48 h. Determination of total biomass was performed by inoculating 10^4^ conidia into ACM broth in pre-weighed, sterile 2 ml microcentrifuge tubes and incubated for 6 h at 37°C at 250 rpm to obtain germinating conidia. Consequently, 25 nM final concentrations of d-siRNAs against target genes were added. After 48 h seeding, reduction in total biomass in d-siRNA treated samples was calculated as follows: percentage of total biomass reduction = 100- (Total biomass in d-siRNA treated lines/ Total biomass in control) X 100.

## Supporting Information

Figure S1 ***A. nidulans*** **s** ***GFP*** **transformants generated on selection medium. (A)** The clear isolated *A. nidulans* colonies were formed on nylon membranes (when *HPT* gene under the control of CaMV35S promoter) placed on selection medium amended with 100 mg/l hygromycin B after 4 days of incubation at 37°C. **(B)** Hygromycin resistant putative fungal transformants were sub-cultured for further growth on ACM amended with 100 mg/l hygromycin B. **(C)** *A. nidulans* colonies were not formed on nylon membranes (*HPT* gene without promoter) placed on selection medium amended with 100 mg/l hygromycin B after 4 days of incubation at 37°C.
(TIF)

Figure S2 **Expression profiles of** ***Ras*** **family genes in un-diced RNA (long dsRNA) treated lines. (A)** Estimation of relative expression levels of *Ras* family genes in untreated control and un-diced An*rasA* dsRNA treated *A. nidulans*. Relative quantification was performed by qRT-PCR using comparative D cycle threshold (CT) method. Expression levels of An*rasB*, An*rhB*, An*4873*, An*medA* and An*7661* in untreated control was showed in the left panel, right panel represents the expression levels of same genes in un-diced An*rasA* dsRNA treated *A. nidulans*. Expression levels of all the genes were normalized to An*Actin* levels. The data represent the means of three replicates. **(B)** Estimation of relative expression levels of *Ras* family genes in untreated control and un-diced An*rasB* dsRNA treated *A. nidulans*. Relative quantification was performed by qRT-PCR using comparative D cycle threshold (CT) method. Expression levels of An*rasA*, An*rhB*, An*4873*, An*medA* and An*7661* in untreated control was showed in the left panel, right panel represents the expression levels of same genes in un-diced An*rasB* dsRNA treated *A. nidulans*. Expression levels of all the genes were normalized to An*Actin* levels. The data represent the means of three replicates. Values were compared using *t* test.
(TIF)

Figure S3 **Phylogenetic analysis of** ***Ras*** **family genes.** Phylogenetic relationship among *A. nidulans Ras* family genes *AnrasA, AnrasB AnrhB, An4873, AnmedA and An7661* (complete ORFs) was calculated by the Clustal-W program.
(TIF)

Figure S4 **Multiple sequence alignment of** ***Ras*** **family genes.** Sequences of *A. nidulans Ras* family genes *AnrasA, AnrasB AnrhB, An4873, AnmedA and An7661* (complete ORFs) were aligned using Clustal-W program.
(RTF)

Figure S5 **Cloning strategy for construction of pCAMBIA1300-s** ***GFP*** **vector.** The s*GFP* gene was PCR amplified from the pMT-sGFP vector and cloned in pGEM T-easy vector. Then, the s*GFP* gene was sub-cloned in pSilent-1 vector in *Xho*I and *Kpn*I restriction sites by removing the spacer DNA and finally expression cassette was introduced into pCAMBIA1300 backbone at the *Xba*I restriction site.
(TIF)

Table S1 **List of primers used in the study.** All the primers used in the study were listed in the table along with their applications. The primers used in qRT-PCR analysis were designed using “Primer Quest” software (Integrated DNA Technologies, USA); remaining primers were designed using Primer3 version v.0.4.0 software.
(DOC)

Table S2 **A: List of siRNA candidates predicted in An** ***rasA*** **target gene.** The partial sequences of the target gene was evaluated for presence of candidate siRNAs using the siRNA selection software. All the identified candidate siRNAs were scrutinized for silencing potency using the standard parameters. List of candidate siRNAs along with their probable mRNA target site and score of each individual siRNA were presented in the table. **B: List of siRNA candidates predicted in An** ***rasB*** **partial nucleotide sequence.** The partial sequences of the target gene was evaluated for presence of candidate siRNAs using the siRNA selection software. All the identified candidate siRNAs were scrutinized for silencing potency using the standard parameters. List of candidate siRNAs along with their probable mRNA target site and score of each individual siRNA were presented in the table. **C: List of siRNA candidates predicted in s** ***GFP*** **nucleotide sequence.** The partial sequences of the target gene was evaluated for presence of candidate siRNAs using the siRNA selection software. All the identified candidate siRNAs were scrutinized for silencing potency using the standard parameters. List of candidate siRNAs along with their probable mRNA target site and score of each individual siRNA were presented in the table.
(DOC)
