# Supplementary figures and images for: Targeting Fungal Genes by Diced siRNAs: A Rapid Tool to Decipher Gene Function in Aspergillus nidulans
Source: PLoS One. 2013 Oct 10;8(10):e75443. doi: 10.1371/journal.pone.0075443 (PMC3794931; doi:10.1371/journal.pone.0075443)

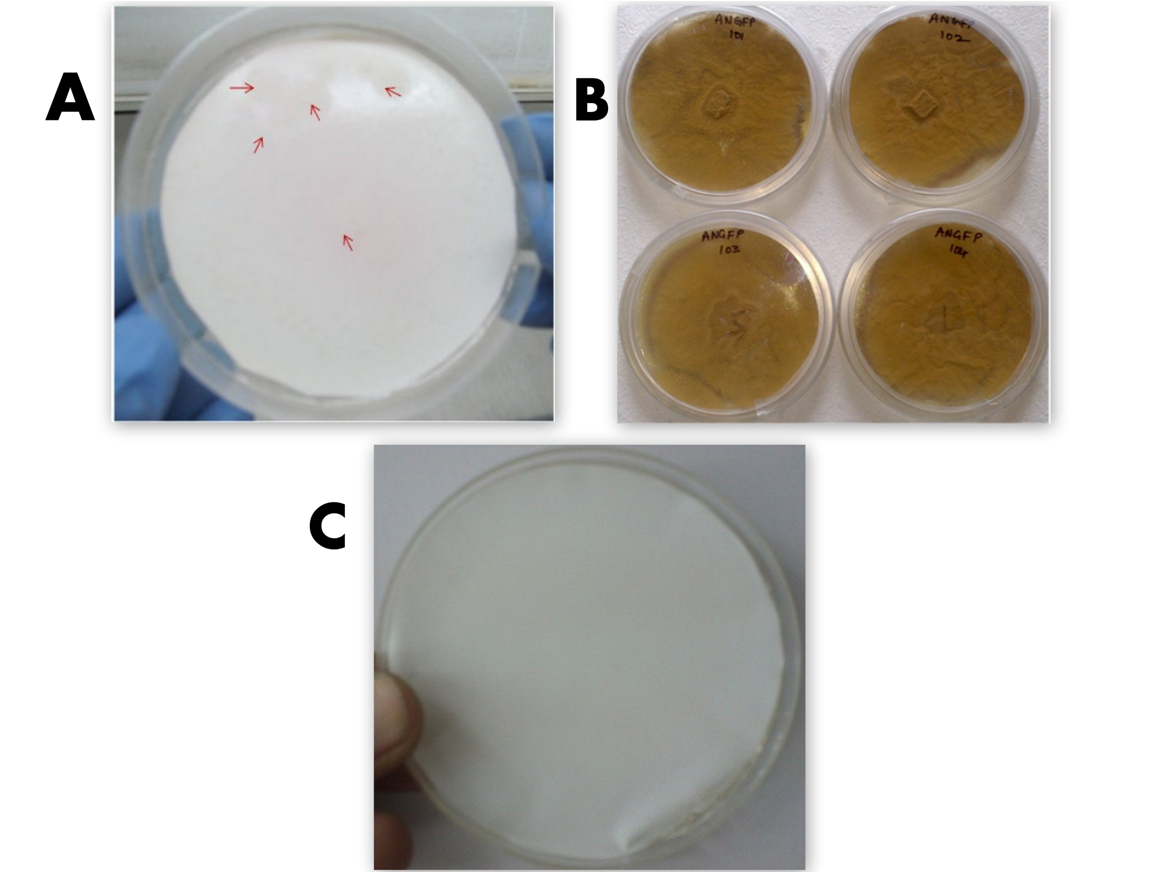

Supplement: Figure S1 — A. nidulans s GFP transformants generated on selection medium. (A) The clear isolated A. nidulans colonies were formed on nylon membranes (when HPT gene under the control of CaMV35S promoter) placed on selection medium amended with 100 mg/l hygromycin B after 4 days of incubation at 37°C. (B) Hygromycin resistant putative fungal transformants were sub-cultured for further growth on ACM amended with 100 mg/l hygromycin B. (C) A. nidulans colonies were not formed on nylon membranes (HPT gene without promoter) placed on selection medium amended with 100 mg/l hygromycin B after 4 days of incubation at 37°C. (TIF) [file pone.0075443.s001.tif]

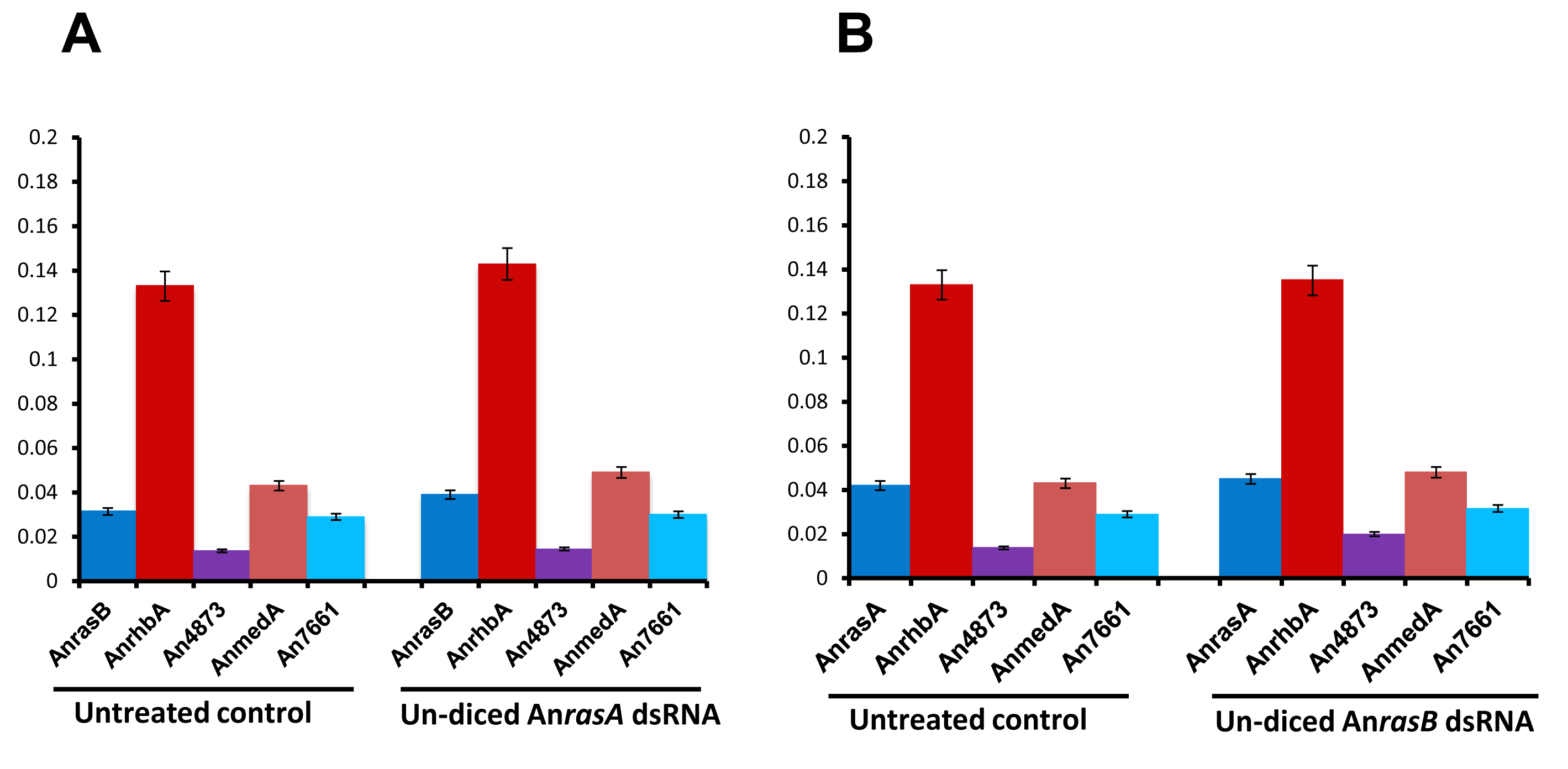

Supplement: Figure S2 — Expression profiles of Ras family genes in un-diced RNA (long dsRNA) treated lines. (A) Estimation of relative expression levels of Ras family genes in untreated control and un-diced AnrasA dsRNA treated A. nidulans. Relative quantification was performed by qRT-PCR using comparative D cycle threshold (CT) method. Expression levels of AnrasB, AnrhB, An4873, AnmedA and An7661 in untreated control was showed in the left panel, right panel represents the expression levels of same genes in un-diced AnrasA dsRNA treated A. nidulans. Expression levels of all the genes were normalized to AnActin levels. The data represent the means of three replicates. (B) Estimation of relative expression levels of Ras family genes in untreated control and un-diced AnrasB dsRNA treated A. nidulans. Relative quantification was performed by qRT-PCR using comparative D cycle threshold (CT) method. Expression levels of AnrasA, AnrhB, An4873, AnmedA and An7661 in untreated control was showed in the left panel, right panel represents the expression levels of same genes in un-diced AnrasB dsRNA treated A. nidulans. Expression levels of all the genes were normalized to AnActin levels. The data represent the means of three replicates. Values were compared using t test. (TIF) [file pone.0075443.s002.tif]

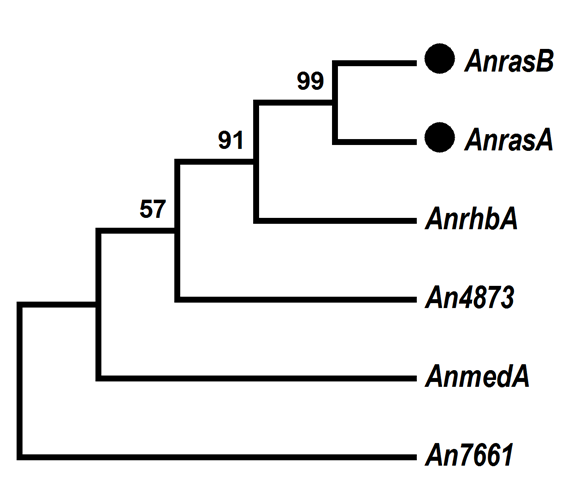

Supplement: Figure S3 — Phylogenetic analysis of Ras family genes. Phylogenetic relationship among A. nidulans Ras family genes AnrasA, AnrasB AnrhB, An4873, AnmedA and An7661 (complete ORFs) was calculated by the Clustal-W program. (TIF) [file pone.0075443.s003.tif]

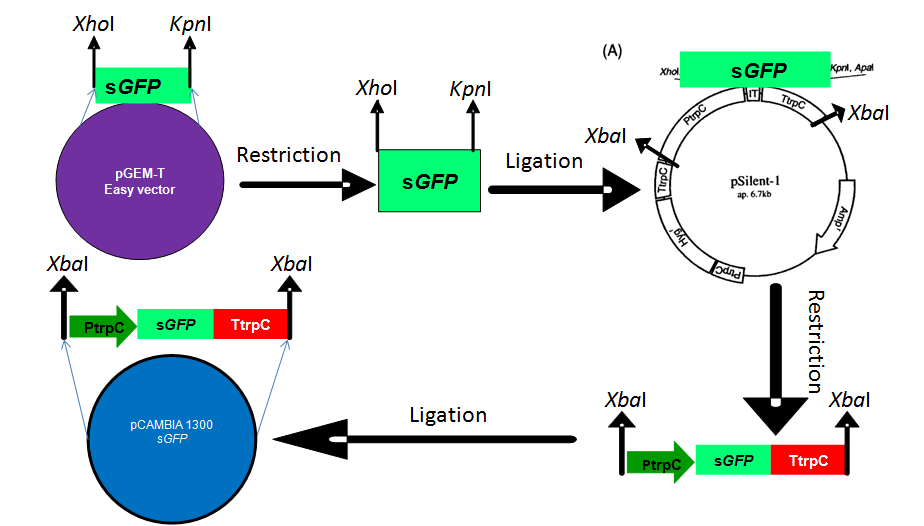

Supplement: Figure S5 — Cloning strategy for construction of pCAMBIA1300-s GFP vector. The sGFP gene was PCR amplified from the pMT-sGFP vector and cloned in pGEM T-easy vector. Then, the sGFP gene was sub-cloned in pSilent-1 vector in XhoI and KpnI restriction sites by removing the spacer DNA and finally expression cassette was introduced into pCAMBIA1300 backbone at the XbaI restriction site. (TIF) [file pone.0075443.s005.tif]
