# Supplementary material for: Targeting Fungal Genes by Diced siRNAs: A Rapid Tool to Decipher Gene Function in Aspergillus nidulans
Source: PLoS One. 2013 Oct 10;8(10):e75443. doi: 10.1371/journal.pone.0075443 (PMC3794931; doi:10.1371/journal.pone.0075443)
Supplement: Figure S4 — Multiple sequence alignment of Ras family genes. Sequences of A. nidulans Ras family genes AnrasA, AnrasB AnrhB, An4873, AnmedA and An7661 (complete ORFs) were aligned using Clustal-W program. (RTF) [file pone.0075443.s004.rtf]

                            10        20        30        40        50        60        70        80        90                 
                   ....|....|....|....|....|....|....|....|....|....|....|....|....|....|....|....|....|....|
AnrasA             ------------------------------------------------------------------------------------------ AnrasA                        
AnrasB             ------------------------------------------------------------------------------------------ AnrasB                        
AnrhbA             ------------------------------------------------------------------------------------------ AnrhbA                        
An7661             ATGGCGGCCCCTAGTATGGGCAGTGATTTCCAACTCTTCTCTCCCGCACAGTCGAGGGGTAGGAAGAGTTCCCAGGGCGACTCCGGCTCG An7661                        
An4873             ------------------------------------------------------------------------------------------ An4873                        
AnmedA             ------------------------------------------------------------------------------------------ AnmedA                        
Clustal Consensus                                                                                             Clustal Consensus             

                           100       110       120       130       140       150       160       170       180        
                   ....|....|....|....|....|....|....|....|....|....|....|....|....|....|....|....|....|....|
AnrasA             ------------------------------------------------------------------------------------------ AnrasA                        
AnrasB             ------------------------------------------------------------------------------------------ AnrasB                        
AnrhbA             ------------------------------------------------------------------------------------------ AnrhbA                        
An7661             GATGATACAGGGCAGGATTGGACGGAATGGATGAGATGGGATGAGCAGGCTTTCCCAGACAACAAGAACTTGCCGTTATCGCCGTCTCTC An7661                        
An4873             ------------------------------------------------------------------------------------------ An4873                        
AnmedA             ------------------------------------------------------------------------------------------ AnmedA                        
Clustal Consensus                                                                                             Clustal Consensus             

                           190       200       210       220       230       240       250       260       270        
                   ....|....|....|....|....|....|....|....|....|....|....|....|....|....|....|....|....|....|
AnrasA             ------------------------------------------------------------------------------------------ AnrasA                        
AnrasB             ------------------------------------------------------------------------------------------ AnrasB                        
AnrhbA             ------------------------------------------------------------------------------------------ AnrhbA                        
An7661             ACATCGCCACCCTTCTCTGATGGGAATAAGAGTATCGACCTCTTCCCATCTGGGGACTTCTCTCCCAGCATTCCCATTGACTATACCTTT An7661                        
An4873             ------------------------------------------------------------------------------------------ An4873                        
AnmedA             ------------------------------------------------------------------------------------------ AnmedA                        
Clustal Consensus                                                                                             Clustal Consensus             

                           280       290       300       310       320       330       340       350       360        
                   ....|....|....|....|....|....|....|....|....|....|....|....|....|....|....|....|....|....|
AnrasA             ------------------------------------------------------------------------------------------ AnrasA                        
AnrasB             ------------------------------------------------------------------------------------------ AnrasB                        
AnrhbA             ------------------------------------------------------------------------------------------ AnrhbA                        
An7661             TCGGCAAACTCTGTTGTATCCGGATCTCCTCTCTCGACAGGCGCTGGGCAAAAGCGTAAGTCAGGGAGCGACGACGACGGATCAGCTGGC An7661                        
An4873             ------------------------------------------------------------------------------------------ An4873                        
AnmedA             ------------------------------------------------------------------------------------------ AnmedA                        
Clustal Consensus                                                                                             Clustal Consensus             

                           370       380       390       400       410       420       430       440       450        
                   ....|....|....|....|....|....|....|....|....|....|....|....|....|....|....|....|....|....|
AnrasA             ------------------------------------------------------------------------------------------ AnrasA                        
AnrasB             ------------------------------------------------------------------------------------------ AnrasB                        
AnrhbA             ------------------------------------------------------------------------------------------ AnrhbA                        
An7661             AGTGGCATGGTACAGGAGGTAAAGAAGGTACCGTCTAAGAAACGAGCCCACAATGTCATCGAGAAGCGATATCGTGCGAACTTGAATGAG An7661                        
An4873             ------------------------------------------------------------------------------------------ An4873                        
AnmedA             ------------------------------------------------------------------------------------------ AnmedA                        
Clustal Consensus                                                                                             Clustal Consensus             

                           460       470       480       490       500       510       520       530       540        
                   ....|....|....|....|....|....|....|....|....|....|....|....|....|....|....|....|....|....|
AnrasA             ------------------------------------------------------------------------------------------ AnrasA                        
AnrasB             ------------------------------------------------------------------------------------------ AnrasB                        
AnrhbA             ------------------------------------------------------------------------------------------ AnrhbA                        
An7661             AAAATAGCAGAGCTTAGGGATAGTGTTCCCAGTCTGCGAGCGTCTAAGGGGAACGGGGTTCTGGACGACGAGGATGAGGGTGTCACGCCG An7661                        
An4873             -----------------------------------------------------------------------------------ATGCTTT An4873                        
AnmedA             ------------------------------------------------------------------------------------------ AnmedA                        
Clustal Consensus                                                                                             Clustal Consensus             

                           550       560       570       580       590       600       610       620       630        
                   ....|....|....|....|....|....|....|....|....|....|....|....|....|....|....|....|....|....|
AnrasA             ------------------------------------------------------------------------------------------ AnrasA                        
AnrasB             ------------------------------------------------------------------------------------------ AnrasB                        
AnrhbA             ------------------------------------------------------------------------------------------ AnrhbA                        
An7661             GCAAACAAACTGAACAAGGCTTCTATTCTTTCAAAAGCGACAGATTACATTCGGCATCTGGAGACCCGAAATAAGCGCCTGGAAGACGAG An7661                        
An4873             CTAATCCACAAAGTACCCTTCACGGGCGCCATCGTCAACATCGACGGCAGATCTCGACTCCCTCCGCGCTCGATGCCGTAAAACCCCCAG An4873                        
AnmedA             ------------------------------------------------------------------------------------------ AnmedA                        
Clustal Consensus                                                                                             Clustal Consensus             

                           640       650       660       670       680       690       700       710       720        
                   ....|....|....|....|....|....|....|....|....|....|....|....|....|....|....|....|....|....|
AnrasA             ------------------------------------------------------------------------------------------ AnrasA                        
AnrasB             ------------------------------------------------------------------------------------------ AnrasB                        
AnrhbA             ------------------------------------------------------------------------------------------ AnrhbA                        
An7661             AATACCGCCTTGAAGGTTAGACTTCGCGAATTGGAAAAGGTGGCCGATCAATCCTTGACATCCGCAGCCTCTGTCTCGTCGCCTAGCAAC An7661                        
An4873             GCCTTTCTCCACAGGCTCTGCAGAGATATCATGCTCATCGCCGCGGCCAAAGTCTGGACCAGCGAGCTGTACAAGCTCAAGCTCAGCGAC An4873                        
AnmedA             ------------------------------------------------------------------------------------------ AnmedA                        
Clustal Consensus                                                                                             Clustal Consensus             

                           730       740       750       760       770       780       790       800       810        
                   ....|....|....|....|....|....|....|....|....|....|....|....|....|....|....|....|....|....|
AnrasA             ------------------------------------------------------------------------------------------ AnrasA                        
AnrasB             ------------------------------------------------------------------------------------------ AnrasB                        
AnrhbA             ------------------------------------------------------------------------------------------ AnrhbA                        
An7661             TATACTGTTTCTACCGAGTCCGCAGGAAGCTCGTCTCCCAGTATTTTCTCGAATCCGGAAGAAAGTCCCATTGAGCCTTCGTCGTCGTCC An7661                        
An4873             AACAGCTCGTGCAAGATGCGTCAAGTACTAACCAAACAGCACCGCAATTCGCGCCTAACTCAACCCTCGTCCCCTTAATTCCTGACTCCC An4873                        
AnmedA             ------------------------------------------------------------------------------------------ AnmedA                        
Clustal Consensus                                                                                             Clustal Consensus             

                           820       830       840       850       860       870       880       890       900        
                   ....|....|....|....|....|....|....|....|....|....|....|....|....|....|....|....|....|....|
AnrasA             ------------------------------------------------------------------------------------------ AnrasA                        
AnrasB             ------------------------------------------------------------------------------------------ AnrasB                        
AnrhbA             ------------------------------------------------------------------------------------------ AnrhbA                        
An7661             TCTCGTCCCGCAGCAGGGATGATTCAGTTACCGGACTCGTTTAAACGCATGCGCACCGAACAATCCAAAGACAACCTTTGGTCGCAGTCT An7661                        
An4873             AGATCTTCGGCCAAGACGACATGCAGGCTTCAAGTCACGCCAATTACCAGACGCCTCACAGCCTACCCTACTTGCACACGAATTTTGTCA An4873                        
AnmedA             -----------------------------------------------ATGTCTGGTTACCAGAGAACCCCACAAGCGGCCGTGCTAGATT AnmedA                        
Clustal Consensus                                                                                             Clustal Consensus             

                           910       920       930       940       950       960       970       980       990        
                   ....|....|....|....|....|....|....|....|....|....|....|....|....|....|....|....|....|....|
AnrasA             ------------------------------------------------------------------------------------------ AnrasA                        
AnrasB             ------------------------------------------------------------------------------------------ AnrasB                        
AnrhbA             ------------------------------------------------------------------------------------------ AnrhbA                        
An7661             TATATGCAGTGTCCGAGTTCAAACAGCGTGTCTACTCAATCTGGAAACGGTCGTCGGCGGTCATACTATCCGAATAAGTACGTTCTCGGT An7661                        
An4873             AGGCCGATGATCAGGCTCGGGATGCTCGACCTGTCAATCACCATCTCAATCTCATTCAACAGCAGCAGCAACAACTGCACAATGCTAAGC An4873                        
AnmedA             GTGATTCTGCCCAATCTCTGCAGGATGGCGCTGCTTATTCTACTTACGGCCAGCCGGTTTATATGTCGACTCCCCTTGCCCCATCTCCAA AnmedA                        
Clustal Consensus                                                                                             Clustal Consensus             

                           1000      1010      1020      1030      1040      1050      1060      1070      1080       
                   ....|....|....|....|....|....|....|....|....|....|....|....|....|....|....|....|....|....|
AnrasA             ------------------------------------------------------------------------------------------ AnrasA                        
AnrasB             ------------------------------------------------------------------------------------------ AnrasB                        
AnrhbA             ------------------------------------------------------------------------------------------ AnrhbA                        
An7661             ACTCTAGCCGGACTTATGGTTTTCGAGGGCCTGGGCAAAGAGAAAGAAACCGACTCAACCGCCAAAGGCTTGTTGGCCATCCCATACAAT An7661                        
An4873             TCAACTGCCACGATACACACGATGATCAGCTGCTCGACAACGACGCGTGGGATACATACAAACCCGACATCGCGTCCTCGCTTCAACAAA An4873                        
AnmedA             TGACCGACCATATAAGCCAGATGTCCGATTGCATGCCATACATGGCGAAGCCTGAGTACGCTAGCTCCTACGAGGATGAGAAGTCGCCAA AnmedA                        
Clustal Consensus                                                                                             Clustal Consensus             

                           1090      1100      1110      1120      1130      1140      1150      1160      1170       
                   ....|....|....|....|....|....|....|....|....|....|....|....|....|....|....|....|....|....|
AnrasA             ------------------------------------------------------------------------------------------ AnrasA                        
AnrasB             ------------------------------------------------------------------------------------------ AnrasB                        
AnrhbA             ------------------------------------------------------------------------------------------ AnrhbA                        
An7661             TACTTCAGGAATGTTGAAGTCCCACCCCTTTTCTATGAGATAATGGGCCGGAGTTTCTGGTCGTCCTGGCATGCGAAAGCCATCCTACAT An7661                        
An4873             CGACCACCGATATGAGACGACAATCTGTCCATTCAAACCCAAGTAGCTCATACCATCCGCACACTCCGAAAAAAACAAACTCACACTACT An4873                        
AnmedA             TGATTACTGTGGAACCTTGCCAGTTGCCCGAAGTCACTTCATACTCACCCCAGCGAGGATCCGAAGGAACGAGGGTTTTTGTGCAGCTGC AnmedA                        
Clustal Consensus                                                                                             Clustal Consensus             

                           1180      1190      1200      1210      1220      1230      1240      1250      1260       
                   ....|....|....|....|....|....|....|....|....|....|....|....|....|....|....|....|....|....|
AnrasA             ------------------------------------------------------------------------------------------ AnrasA                        
AnrasB             ------------------------------------------------------------------------------------------ AnrasB                        
AnrhbA             ------------------------------------------------------------------------------------------ AnrhbA                        
An7661             TTCCTCTTCCTTGCTGTTCTCGTTGTTGGGTCAGCTTTCATTGTGTTCGTATATCTGTTCAACTCAGGTCCGGGGCACCAGAACTCGTCC An7661                        
An4873             TCCCAATCTCCCCAGCAACGACGCCGTTCGACAAAACAGATTTTGCTCAGTACTGCGCGGAGACGCAAATCGTCCCAGCAAAAGACCAAA An4873                        
AnmedA             AGTCACCCTACGACCTCCACACAACCCCCTATGCAACACTCTACATCGTGTTCGGATCAAAAAAGTGCGAGTGCAATCCGCACTTTCTCG AnmedA                        
Clustal Consensus                                                                                             Clustal Consensus             

                           1270      1280      1290      1300      1310      1320      1330      1340      1350       
                   ....|....|....|....|....|....|....|....|....|....|....|....|....|....|....|....|....|....|
AnrasA             ------------------------------------------------------------------------------------------ AnrasA                        
AnrasB             ------------------------------------------------------------------------------------------ AnrasB                        
AnrhbA             ------------------------------------------------------------------------------------------ AnrhbA                        
An7661             AAGCTGTCAACCCCAGGTGTCA--TGCTCTCCTCGTCAAATTTCAGACGCCAAGCGTGGTTGACCAGCATCCAGCGAGTTGGGGTCCCAA An7661                        
An4873             ATGCTGCTGATGCCAGCTCCCAGTCGGCCTATATGCAACGCGCCAAGTCCCTTCAAGGAGTAGCGGGGACTAGCTTCTCACAGCAAAAGA An4873                        
AnmedA             GATTCCGGGACTCTGCCTTCCAATATGCTCTCTCCGTCGACACGCCGCCATTTATGTCTACGGGGTCGCCCTCCCTGGCTGTTCCTCTGC AnmedA                        
Clustal Consensus                                                                                             Clustal Consensus             

                           1360      1370      1380      1390      1400      1410      1420      1430      1440       
                   ....|....|....|....|....|....|....|....|....|....|....|....|....|....|....|....|....|....|
AnrasA             ------------------------------------------------------------------------------------------ AnrasA                        
AnrasB             ------------------------------------------------------------------------------------------ AnrasB                        
AnrhbA             ------------------------------------------------------------------------------------------ AnrhbA                        
An7661             GGCATCGGTTTTTCCACGAATGGTATGTCGTGACTTCACGTTGTTTTGAATACGTTCTACGGTGTCTGCTAGGATGGAAGCTATATTCCT An7661                        
An4873             TTGAAATGCCCTCTCCCCCTAGCACTGATTCGTTTGCAGTTGATGGTTTTGATACGTTTGACTACCAGCAGTGTTCCAGTTTTGATAACC An4873                        
AnmedA             AACTAGCGATGGGCGGTCAAAATGAAAGCCCTGCCACAACTCTGCAGGTGGGCGTTTATACATATGAGAATGCAGGACAGCAGTCGCCCT AnmedA                        
Clustal Consensus                                                                                             Clustal Consensus             

                           1450      1460      1470      1480      1490      1500      1510      1520      1530       
                   ....|....|....|....|....|....|....|....|....|....|....|....|....|....|....|....|....|....|
AnrasA             ------------------------------------------------------------------------------------------ AnrasA                        
AnrasB             ------------------------------------------------------------------------------------------ AnrasB                        
AnrhbA             ------------------------------------------------------------------------------------------ AnrhbA                        
An7661             CCATCACTGGCATCACGGAAGAGGATGAGAAGGGCCGCGTTAAAACTTGGGACATTGCAATTGACGCACAACTCTCTGGCGGAGATGCTG An7661                        
An4873             TCGCTACCACCAGCCACAGCCAGTACTCTACGTCGTCCAACTCACCAGAAGTCGCTGCCATTCCAAGCTCTGGAGATCACACCGAAAAGA An4873                        
AnmedA             CTGAAGATGGCCGGAAGAGGCGAATTTCTTCCTTCTCTGCAGATAGTACCTCCAGGCCGATTAAGCGGGCGTCAACCCTTCCGGTGCAGA AnmedA                        
Clustal Consensus                                                                                             Clustal Consensus             

                           1540      1550      1560      1570      1580      1590      1600      1610      1620       
                   ....|....|....|....|....|....|....|....|....|....|....|....|....|....|....|....|....|....|
AnrasA             ------------------------------------------------------------------------------------------ AnrasA                        
AnrasB             ------------------------------------------------------------------------------------------ AnrasB                        
AnrhbA             ------------------------------------------------------------------------------------------ AnrhbA                        
An7661             AAATCAGCAAAAGCCGACTGGTGCTTACGATCTTCGCTGCAGGAACTTTACCGCGGAGTCCCATGAGGATGATGCTTAAGGCACTCCACT An7661                        
An4873             AGTCCAAGCTCCCTATTTGTCCTGCCACGCCCAGCCGTCTCAGCCCAAGGAAACAGCTCGCTACGCCAAGCGCGGCTTCTTTAGTGAAGG An4873                        
AnmedA             TCAAGGAGGAACAATCTAGCTACGCTGCGCCTTACTCGCCATATCTGCAGCCACTTCCTTCCATGAACGGATTTGCCGCCTCGTATCACA AnmedA                        
Clustal Consensus                                                                                             Clustal Consensus             

                           1630      1640      1650      1660      1670      1680      1690      1700      1710       
                   ....|....|....|....|....|....|....|....|....|....|....|....|....|....|....|....|....|....|
AnrasA             ------------------------------------------------------------------------------------------ AnrasA                        
AnrasB             ------------------------------------------------------------------------------------------ AnrasB                        
AnrhbA             ------------------------------------------------------------------------------------------ AnrhbA                        
An7661             GCCGCATCCTACTATGGAGAGTCGGTG-----TTCCCGGTCAGTGGAGCTATCGAGTGTCTAACGATGTTGCCCGTTCGCTTGCGAAATA An7661                        
An4873             CAAAACTTTCTCCGCGTGTCGCATCTA-----TCGATAACCTCAACCTGGACTCCCGGGTGCATGCCTCTATCAAAGAAACTGGTGTTAG An4873                        
AnmedA             CGGATTCTTCGCCCCGGATGGGTGCTACACAATATACAACCGTATCGACAAACTCGCAGCCTTCGATTCGTGCCCCGTCTCCCATGGCCC AnmedA                        
Clustal Consensus                                                                                             Clustal Consensus             

                           1720      1730      1740      1750      1760      1770      1780      1790      1800       
                   ....|....|....|....|....|....|....|....|....|....|....|....|....|....|....|....|....|....|
AnrasA             ------------------------------------------------------------------------------------------ AnrasA                        
AnrasB             ------------------------------------------------------------------------------------------ AnrasB                        
AnrhbA             ------------------------------------------------------------------------------------------ AnrhbA                        
An7661             TCAGTGGGACCTGGCACGGAAGATGAATGCAGCACTGCCAAAAGACCACGAGGATGCACTTCCGCCCCATCTCGAAGCTTTACTTCAGTG An7661                        
An4873             CATTGATGAAATAGCGTCCTATATCCACGGTCCAGACCCCGAAGACGGAAAGTGGGTGTGCCTGCACCCCGGCTGTGAGCGACGCTTTGG An4873                        
AnmedA             CGTCGCCAATGGCACCTTCGTGGAACTCGTCTTTCCTCTCGGTCAACCATGATCAGAGAGGTTCCGGGTATGCAGTCGGCCGTGGCGTTT AnmedA                        
Clustal Consensus                                                                                             Clustal Consensus             

                           1810      1820      1830      1840      1850      1860      1870      1880      1890       
                   ....|....|....|....|....|....|....|....|....|....|....|....|....|....|....|....|....|....|
AnrasA             ------------------------------------------------------------------------------------------ AnrasA                        
AnrasB             ------------------------------------------------------------------------------------------ AnrasB                        
AnrhbA             ------------------------------------------------------------------------------------------ AnrhbA                        
An7661             TGAAAGTGAGGACGTGATGATCGATAGCATCACTCAAAGGGCGGCCAATTTGACCTGGAATGATCCTACACAGGAAGGATCCGATGGCGA An7661                        
An4873             CCGCAAGGAAAACATCAAGTCACATGTGCAAACCCACCTAGGTGATCGCCAGTACAAGTGCGATCATTGTGATAAGTGTTTCGTTCGTGG An4873                        
AnmedA             GCCAGCCAAAGCCCTCGTCACCTGCGAGCTTTTCGAACCCAACTCTCATCCGTACCTCCACATTGCAGCAGTCGGCTGGTGTCGTCCAAA AnmedA                        
Clustal Consensus                                                                                             Clustal Consensus             

                           1900      1910      1920      1930      1940      1950      1960      1970      1980       
                   ....|....|....|....|....|....|....|....|....|....|....|....|....|....|....|....|....|....|
AnrasA             ------------------------------------------------------------------------------------------ AnrasA                        
AnrasB             ------------------------------------------------------------------------------------------ AnrasB                        
AnrhbA             ------------------------------------------------------------------------------------------ AnrhbA                        
An7661             CGACGCTTTTCTGGACGTGGTGGAAGAGGATCCGGCAATCCAGTCTTCTCTAGATGCACTTGCGGCATGGTGGTCCTCTCACCTTCTACA An7661                        
An4873             GCATGATCTGAAGCGCCACGCGAAGATCCACACAGGAGACAAGCCGTACGAATGTCTATGCGGTAATGTTTTTGCCCGGCACGATGCCCT An4873                        
AnmedA             CACAATCGTTCAACCCCTATGCCATGTATCCGTCAAAGGCAGTATTGAAGCTCAATGGTGACCTAAATACCATGACGCATAATTGGACTC AnmedA                        
Clustal Consensus                                                                                             Clustal Consensus             

                           1990      2000      2010      2020      2030      2040      2050      2060      2070       
                   ....|....|....|....|....|....|....|....|....|....|....|....|....|....|....|....|....|....|
AnrasA             --------------------------------------------------------------------------------------ATGG AnrasA                        
AnrasB             --------------------------------------------------------------------------------------ATGG AnrasB                        
AnrhbA             --------------------------------------------------------------------------------------ATGC AnrhbA                        
An7661             GCGAGCTCTTCTCAAGTATTTTGAGGCTAGTGCTCGCGGACCGGATGCAAAAAAGAGCCGTGACATGTTCAAGGCTAAAATTCAACTCGC An7661                        
An4873             AACTCGGCACCGCCAGAGGGGAATGTGCATTGGTGGTTACAAGGGAATTGTGCGTAAGACAACGAAACGTGGCCGTCCTAAGAAGCACCG An4873                        
AnmedA             GGGAGGAGCAAGTTGCTCAGCGCCGCCTGGTTCAATTTACACGAATGCAAAGTGGCAGCACTATTCACGCTGATTTCAAGCCTGTGTCTC AnmedA                        
Clustal Consensus                                                                                             Clustal Consensus             

                           2080      2090      2100      2110      2120      2130      2140      2150      2160       
                   ....|....|....|....|....|....|....|....|....|....|....|....|....|....|....|....|....|....|
AnrasA             CTTCAAAGTTTCTAC--GAGAG--TACAAGCTGGTCGTCGTCG-GTGGTGGTGGTGTTGGAAAGTCATGCTTGACG-ATTCAATTGATCC AnrasA                        
AnrasB             CCGGAAAAATGACTC--TC-----TATAAATTGGTGGTGCTGG-GAGATGGTGGTGTCGGGAAGACTGCTCTCACA-ATTCAGTTGTGTC AnrasB                        
AnrhbA             CTAGTGCGCCAAAAC--AA-----CGCAAGATAGCTATTGTCG-GCAGTCGCTCCGTGGGCAAATCCTCACTTACG-GTTCGGTTTGTTG AnrhbA                        
An7661             TCTTGACGTGGCACC--ACAGC--CTTCTGCCGCTCATACCCGCGCCCTGGTCATGATGGCG-GTGTTCTTTGA---GCAGGACCGGGTC An7661                        
An4873             CCCAGAGATGGATGA--GAGACGTGACAAGGCAACCAAGACCCGACAGAGG-ATCGCTGAGAAATCATTATTCA---ATTCTTCCGAATC An4873                        
AnmedA             CCGAAGAGCGGGCTCCGAACAGCATTTGTATCAGCTGCATTTA--TTGGGAAGGCAAGGATGAGTGTTTCATCACGAGTGTGGACACTAT AnmedA                        
Clustal Consensus                                                            *            * *                 Clustal Consensus             

                           2170      2180      2190      2200      2210      2220      2230      2240      2250       
                   ....|....|....|....|....|....|....|....|....|....|....|....|....|....|....|....|....|....|
AnrasA             AGAGCCACTTC-GTGGACGAATATGACCCAA----CAATTGAAGATTCATACCGCAAGCAGTGTGTCATTGACGA--TGAGGTCGCTCTG AnrasA                        
AnrasB             TAAACCATTTC-GTCGAAACATACGATCCGA----CCATTGAAGATTCGTATCGCAAGCAAGTCGTCATTGATCA--GCAGTCATGCATG AnrasB                        
AnrhbA             AACATCATTTT-GTGGAGAGTTATTACCCGA----CGATTGAGAACACCTTCAGTCAAATCATCAAATACAACGG--TCAGGATTATGCA AnrhbA                        
An7661             AAGAACATTG----GGGCCGTTCTTGCCGCA----CTGCCAAAGGAGAAGTCCAAAAGCAAACAGAACCAGACGT--TCAACTTTCTGGA An7661                        
An4873             GGACACTTCTC-GTCGTACGCCGCCCTCGGAG--GTGTTTGAGAACATGAGCCTTCATGGCTCCAGCTCAGCAGA--CGAGATGGTGACA An4873                        
AnmedA             CTATCTACTTGAGTCGCTCGTTGGAGTCCGTTTTACGGTGGAGGAGAAGAATCGTATTCGACGAAACCTGGAAGGCTTTAGACCTTTAAC AnmedA                        
Clustal Consensus                 *           *             *                                     *           Clustal Consensus             

                           2260      2270      2280      2290      2300      2310      2320      2330      2340       
                   ....|....|....|....|....|....|....|....|....|....|....|....|....|....|....|....|....|....|
AnrasA             TTGGATGTCCTCGATA-CCGCCGGACAGGAA--------GAATACTCTGCCATGCGCGAA--CAATATATGCGAACGGGCGAAGGCTTCC AnrasA                        
AnrasB             TTGGAGGTCCTCGATA-CTGCCGGCCAGGAG--------GAGTACACCGCACTACGCGAC--CAATGGATCCGGGACGGCGAGGGGTTCG AnrasB                        
AnrhbA             ACAGAGATAGTTGATA-CAGCCGGTCAAGAT--------GAGTACAGTATATTGAACTCA--AAACATTTCATCGGGATTCATGGATATA AnrhbA                        
An7661             TTCGTCACTCCCGGTATCTGTCCGCGAGGAG--------ATTTCTATCGCAGTTCGTTGCGCCATGATTGCCGCGATATTCACCGCACGG An7661                        
An4873             TTTGACAGCCAAAATTACTTGCCGCCAGAAG--------TGTTCACTTTCACTCCGCCCG---AATCTCCAAATTACGGTACAGCAAGCA An4873                        
AnmedA             TGTGTCCAAATCCAAAGCAGACAGCGAAGAGTTCTTCAAGGTTATCATGGGATTCCCTGCCCCGA-AGCCGAGAAACATTGAAAAGGATG AnmedA                        
Clustal Consensus     *             *   * *  *  *            *         *                                      Clustal Consensus             

                           2350      2360      2370      2380      2390      2400      2410      2420      2430       
                   ....|....|....|....|....|....|....|....|....|....|....|....|....|....|....|....|....|....|
AnrasA             TCCTGGTCTACTCCATCACATCGCG--TCAAT--CCTTTGAAGAAATCAT----GACATT-CCAACAACAAATC---------------- AnrasA                        
AnrasB             TCCTCGTCTACAGCATTACGTCGCG--TGCTT--CCTTCTCCAGAATAAC----GAAGTT-CTACAACCAAATCAAGATGGTCAAGGAGT AnrasB                        
AnrhbA             TTATTGCCTATTCCGTGGCGTCCCG--CCAAT--CTTTTGATATGGTAAG----GGTCAT-CAGGGACAAGATA---------------- AnrhbA                        
An7661             TCCCGCCATGACAATTCACTTCCCGAGTCATTTACCATGGAGAAAGCCGT----GACTTG-GTTTGATCAGCTGCCGCTGGATCCCGTTG An7661                        
An4873             AGCCTGCC-AGCCCGCGATCTCTCACGCCGAGCTCCGAAGACGAGATGCT----ACCTTTGTCATCATCCAAACGACCACTGGAAAACAT An4873                        
AnmedA             TCAAAGTCTTTCCTTGGAAGATTTTAGGCCAT-GCTTTGAAAAAGATCATCGGCAAATATTCTGCAAGCTATTCATCTACCGCTGGGGCT AnmedA                        
Clustal Consensus                                    *                               *                        Clustal Consensus             

                           2440      2450      2460      2470      2480      2490      2500      2510      2520       
                   ....|....|....|....|....|....|....|....|....|....|....|....|....|....|....|....|....|....|
AnrasA             ----------------------------------------CTCCGCGTTAAGGACA-----AGGACTACT--------TCCCTATCATTG AnrasA                        
AnrasB             CTGCGAATTCCAGCTCGCCGTCTGGCGCCAGCTATCTAGGCTCCCCGATGAGCTCGCCCTCAGGGCCACCACTTCCAGTTCCCGTAATGC AnrasB                        
AnrhbA             -------------------------------------------CTGAATCATCTCGGA--GCGGACTATG--------TTCCCCTGGTCG AnrhbA                        
An7661             ATTTGACACTTCTTGGTTTCTCCGCTGTCTACCACTTGCTTCACGTCCTCGCATCGGA-TACGGGCTACTTGTCGTCGTCGGACTCGTCG An7661                        
An4873             TCTTGAGCATTCGGGCCTCCCC------------------CTTCTCACTGATGCCGGCACATGCTCTTTCTCCTCTGTTTCAAGTTCAAG An4873                        
AnmedA             CTGCCCACACCCATTGGGTCCAACTACCCAAGTACT--GGCCCCGCATCTGACTCTGG-CGCAGAAGGTCAAAGCGCTGCCTCTCCCCAG AnmedA                        
Clustal Consensus                                             *          *                                    Clustal Consensus             

                           2530      2540      2550      2560      2570      2580      2590      2600      2610       
                   ....|....|....|....|....|....|....|....|....|....|....|....|....|....|....|....|....|....|
AnrasA             TCGTCGGCAACAAATGCGATCTCGACAAGGA---GCGAGTGGTATCCGAACAAGAGGGCGAATCCTTGGCACGACAGTTCGGCTGCAAGT AnrasA                        
AnrasB             TGGTCGGAAACAAGAGTGACAAAGCGGTTGA---GCGGGCGGTTTCGGCCCAGGAAGGCCAGGCGCTTGCTAAAGACCTAGGATGCGAGT AnrasB                        
AnrhbA             TTGTGGGGAATAAGAGTGACCTCAAGCCTGAACAGCGCCAAGTTTCCTTAGACGAAGGCCGACAGTTAGGTGAAGAGTTTCGCTGTGCAT AnrhbA                        
An7661             CGACCATCCTCACCTATGCTTAAAGATTCAAC--TCTTGGAGATTCGTCCGATGATG-CGGAAGATGAGGCAGAAGAATCAATTACGCAC An7661                        
An4873             CAGCCATGCACTATCTTCTCCGCACACCGCGCCTACCCTAAGCGACCCTTCGCAACCATCCGATCTC-GATATCTTCATCAACAGTGAAC An4873                        
AnmedA             TCGATATCCGAAGGGACGCCCTCGAGCAGCTATCACCAGAGCAATGCAGTCCCAATTTACTCGCCCCCGACTGAAACTGACGG-ACCAAT AnmedA                        
Clustal Consensus                                     *                  *             *                      Clustal Consensus             

                           2620      2630      2640      2650      2660      2670      2680      2690      2700       
                   ....|....|....|....|....|....|....|....|....|....|....|....|....|....|....|....|....|....|
AnrasA             TCATCGAAACATCCGCGAAATCGCGCATCAAT-GTCGAAAACGCTTTTTACGACCTTGTGCG-TGAGATCCGCCG-------GTACAA-- AnrasA                        
AnrasB             TTGTCGAGGCATCCGCGAAGAACTGCATCAAC-GTTGAGAAGGCGTTTTACGATGTCGTCAG-AATGCTGCGCAGCCAGCGCATGCAA-- AnrasB                        
AnrhbA             TTACGGAAGCTAGTGCTCGTCTTGATTTCAAC-GTTGCGAGAGCTTTCGAGTTGATGATTGG-GGAAATTGAAAA---------ATCC-- AnrhbA                        
An7661             AGGAAAGAAACTGCGCCTGAGCCTGTACCGCTTATCGGTCGAGTTGCGTCTGAGCTCATGTA-CTGGGCTCGCAATGCCTACAATCCAAC An7661                        
An4873             CTTCCTCTGCCTTTGGCAAACAAGATTTCGGC--TTGGGTGATTCGGACATGGCTGCATTCC-CAGACTACGTCAACGGC-TCTGCGT-T An4873                        
AnmedA             TCGAACAATTCTACCCGCCGTCAGCCAGTCATACTCAAATATGACTGCACCGTACTCTTATACTACGGTCTGCCACT----CAGGTCAGG AnmedA                        
Clustal Consensus                                    *                       *                                Clustal Consensus             

                           2710      2720      2730      2740      2750      2760      2770      2780      2790       
                   ....|....|....|....|....|....|....|....|....|....|....|....|....|....|....|....|....|....|
AnrasA             CAAGGAGATGTCCAACCCAT-----------CTGGATCCGGT-GCATTCGGCGCCCGCGCCCCCGACAGCAAAATGGACGTAAGCGAG-C AnrasA                        
AnrasB             CAGCAAAGACCCCAGGACACTCGCCGGACGACTGGTCTTGGCCAGATGCGTGACTCTGGCCCCGAGTACCCCAGGACATTTCGCCCCG-A AnrasB                        
AnrhbA             CAGAATCCATCTCAACCAACTGGTGGT---AACAAATGTGCTTTAATGTGA--------------------------------------- AnrhbA                        
An7661             CTTCTATGGCTTCACCTCAGATCTTGTTCGAGTCATCGAGGAAGAATGTACTTCTCTATGCCACGGCGCTGGAGTCGACGTTGCAGACTA An7661                        
An4873             TGACAGCAGCTTGGATTTGCTCCAAGG---GAAGAATTTCTCCACAGGGCCCTCTATGGGCGATGACTTCTTTTCCTTCCAGTTCCAAGT An4873                        
AnmedA             TTGCACCCAGCGGCCCTGCAGCGAGGAGTTGGGAGCTCAACCAGCTTGCCACTCCTTCAACCATGAATGGACACCCGAATCCTGGCAATT AnmedA                        
Clustal Consensus                                                                                             Clustal Consensus             

                           2800      2810      2820      2830      2840      2850      2860      2870      2880       
                   ....|....|....|....|....|....|....|....|....|....|....|....|....|....|....|....|....|....|
AnrasA             CTGGTGAGA-GCGCCGGCTGCTGCGGGAAATGTATTGTTATGTAA--------------------------------------------- AnrasA                        
AnrasB             CCGCCCTCG-ACACCG-CGGCGGTATCAAGTGTCAAATCCTGTGA--------------------------------------------- AnrasB                        
AnrhbA             ------------------------------------------------------------------------------------------ AnrhbA                        
An7661             CTCCCGACT-TCATCAGGAAAGGTTGAAGGCGAGTCGTCGCAAAGATAAACGAAAGGCCAAATCACGGTTGTCAAAAGAACGCGCTACGC An7661                        
An4873             CGACGAACAAGCGTCGGACGTCATGTCAAGGGAGTTTTTCCTCGACTAA----------------------------------------- An4873                        
AnmedA             TCAATTACATGCCTCCGATGACTTATTCTCAGTCTGATTACCCCCAGGGACACTAG---------------------------------- AnmedA                        
Clustal Consensus                                                                                             Clustal Consensus             

                           2890      2900      2910      2920      2930      2940      2950      2960      2970       
                   ....|....|....|....|....|....|....|....|....|....|....|....|....|....|....|....|....|....|
AnrasA             ------------------------------------------------------------------------------------------ AnrasA                        
AnrasB             ------------------------------------------------------------------------------------------ AnrasB                        
AnrhbA             ------------------------------------------------------------------------------------------ AnrhbA                        
An7661             CGCCTGCTGAAACTCATCTGGCTGCAAAGCCAGCGGCTAGTGCTAGTCCAAGTGACTTTCCATGCCCCCTAAAAGACCAACCTGCGCTTG An7661                        
An4873             ------------------------------------------------------------------------------------------ An4873                        
AnmedA             ------------------------------------------------------------------------------------------ AnmedA                        
Clustal Consensus                                                                                             Clustal Consensus             

                           2980      2990      3000       
                   ....|....|....|....|....|....|....|...
AnrasA             --------------------------------------                                                     AnrasA                        
AnrasB             --------------------------------------                                                     AnrasB                        
AnrhbA             --------------------------------------                                                     AnrhbA                        
An7661             TGGGAGAGTCCATTGCAGCGGGGAGGGAACGGACCTAG                                                     An7661                        
An4873             --------------------------------------                                                     An4873                        
AnmedA             --------------------------------------                                                     AnmedA                        
Clustal Consensus                                                                                             Clustal Consensus             
