# Supplementary material for: Targeting Fungal Genes by Diced siRNAs: A Rapid Tool to Decipher Gene Function in Aspergillus nidulans
Source: PLoS One. 2013 Oct 10;8(10):e75443. doi: 10.1371/journal.pone.0075443 (PMC3794931; doi:10.1371/journal.pone.0075443)
Supplement: Table S1 — List of primers used in the study. All the primers used in the study were listed in the table along with their applications. The primers used in qRT-PCR analysis were designed using “Primer Quest” software (Integrated DNA Technologies, USA); remaining primers were designed using Primer3 version v.0.4.0 software. (DOC) [file pone.0075443.s006.doc]

**Table S1. List of primers used in the study.** All the primers used in the study were listed in the table along with their applications. The primers used in qRT-PCR analysis were designed using “Primer Quest” software (Integrated DNA Technologies, USA); remaining primers were designed using Primer3 version v.0.4.0 software.

| **Primer** | **Sequence (5´ to 3´)** | **Application** |
| --- | --- | --- |
| AnrasA-F  AnrasA-R  AnrasB-F  AnrasB-R  sGFP-F  sGFP-R  Hpt-F  Hpt-R  sGFP-sq-F  sGFP-sq-R  T7rasA-F  T7rasA-R  T7rasB-F  T7rasB-R  T7sGFP-F  T7sGFP-R  M 13-F  M 13-R  Actin-RT-F  Actin-RT-R  AnrasA-RT-F  AnrasA-RT-R  AnrasB-RT-F  AnrasB-RT-R  sGFP-RT-F  sGFP-RT-R  Anrhb-RT-F  Anrhb-RT-R  An4873-RT-F  An4873-RT-R  AnmedA-RT-F  AnmedA-RT-R  An7661-RT-F  An7661-RT-R | GGTACCCTCGAGTCAATTGATCCAGAGCC  AGATCTAAGCTTGTTCGGATACCACTCGC  GGTACCCTCGAGAACATACGATCCGACC  AGATCTAAGCTTGTCACTCTTGTTTCCG  CTCGAGAGTGGTTGATGGGCTGCAGG  GGTACCTGACCATGATTACGCCAAGC  ATCGCCTCGCTCCAGTCAATG  AGCTGCGCCGATGGTTTCTACAA  GCAAGCTGACCCTGAAGTT  CCGTCGTCCTTGAAGAAGAT  TAATACGACTCACTATAGGGTCAATTGATCCAGAGCC TAATACGACTCACTATAGGGGTTCGGATACCACTCGC  TAATACGACTCACTATAGGGAACATACGATCCGACC  TAATACGACTCACTATAGGGTGTCACTCTTGTTTCCG  TAATACGACTCACTATAGGGAGTGGTTGATGGGCTGCAGG  TAATACGACTCACTATAGGGTGACCATGATTACGCCAAGC  GTAAAACGACGGCCAGT  CAGGAAACGGCTATGAC  GTATCCACGTCACCACTTTCA  TCTGCATACGGTCGGAGATA  GAACAAGAGGGCGAATCCTT  CTCACGCACAAGGTCGTAAA  AGAGTGACAAAGCGGTTGAG  CAACGTTGATGCAGTTCTTCG  GCACAAGCTGGAGTACAACTA  TGTTGTGGCGGATCTTGAA  ATCATCTCGGAGCGGACTAT  CAGCGAAACTCTTCACCTAACT  TGAACCTTCCTCTGCCTTTG  TCTTCCCTTGGAGCAAATCC  TATACTACGGTCTGCCACTCA  GGATTCGGGTGTCCATTCAT  CGGAAGATGAGGCAGAAGAA  ACGCAACTCGACCGATAAG | Cloning  Screening of fungal transformants  dsRNA synthesis  qRT-PCR |
