# Supplementary material for: Targeting Fungal Genes by Diced siRNAs: A Rapid Tool to Decipher Gene Function in Aspergillus nidulans
Source: PLoS One. 2013 Oct 10;8(10):e75443. doi: 10.1371/journal.pone.0075443 (PMC3794931; doi:10.1371/journal.pone.0075443)
Supplement: Table S2 — A: List of siRNA candidates predicted in An rasA target gene. The partial sequences of the target gene was evaluated for presence of candidate siRNAs using the siRNA selection software. All the identified candidate siRNAs were scrutinized for silencing potency using the standard parameters. List of candidate siRNAs along with their probable mRNA target site and score of each individual siRNA were presented in the table. B: List of siRNA candidates predicted in An rasB partial nucleotide sequence. The partial sequences of the target gene was evaluated for presence of candidate siRNAs using the siRNA selection software. All the identified candidate siRNAs were scrutinized for silencing potency using the standard parameters. List of candidate siRNAs along with their probable mRNA target site and score of each individual siRNA were presented in the table. C: List of siRNA candidates predicted in s GFP nucleotide sequence. The partial sequences of the target gene was evaluated for presence of candidate siRNAs using the siRNA selection software. All the identified candidate siRNAs were scrutinized for silencing potency using the standard parameters. List of candidate siRNAs along with their probable mRNA target site and score of each individual siRNA were presented in the table. (DOC) [file pone.0075443.s007.doc]

**Table S2A. List of siRNA candidates predicted in An*rasA* target gene.** The partial sequences of the target gene was evaluated for presence of candidate siRNAs using the siRNA selection software. All the identified candidate siRNAs were scrutinized for silencing potency using the standard parameters. List of candidate siRNAs along with their probable mRNA target site and score of each individual siRNA were presented in the table.

| **S.No.** | **siRNA candidate** | **Probable mRNA target site** | **Score** |
| --- | --- | --- | --- |
| 1 | CCAUGCGCGAACAAUAUAU UU  UU GGUACGCGCUUGUUAUAUA | TG CCATGCGCGAACAATATAT GC | 7 |
| 2 | GGACGAAUAUGACCCAACA UU  UU CCUGCUUAUACUGGGUUGU | GT GGACGAATATGACCCAACA AT | 7 |
| 3 | GUCAAUCCUUUGAAGAAAU UU  UU CAGUUAGGAAACUUCUUUA | GC GTCAATCCTTTGAAGAAAT CA | 6 |
| 4 | CCCAACAAUUGAAGAUUCA UU  UU GGGUUGUUAACUUCUAAGU | GA CCCAACAATTGAAGATTCA TA | 7 |
| 5 | CGUCAAUCCUUUGAAGAAA UU  UU GCAGUUAGGAAACUUCUUU | CG CGTCAATCCTTTGAAGAAA TC | 6 |
| 6 | CUGGUCUACUCCAUCACAU UU  UU GACCAGAUGAGGUAGUGUA | TC CTGGTCTACTCCATCACAT CG | 5 |
| 7 | GCGCGAACAAUAUAUGCGA UU  UU CGCGCUUGUUAUAUACGCU | AT GCGCGAACAATATATGCGA AC | 3 |
| 8 | CGGACAGGAAGAAUACUCU UU  UU GCCUGUCCUUCUUAUGAGA | GC CGGACAGGAAGAATACTCT GC | 3` |
| 9 | CAUCGCGUCAAUCCUUUGA UU  UU GUAGCGCAGUUAGGAAACU | CA CATCGCGTCAATCCTTTGA AG | 6 |
| 10 | GCGUUAAGGACAAGGACUA UU  UU CGCAAUUCCUGUUCCUGAU | CC GCGTTAAGGACAAGGACTA CT | 5 |
| 11 | CGCGAACAAUAUAUGCGAA UU  UU GCGCUUGUUAUAUACGCUU | TG CGCGAACAATATATGCGAA CG | 5 |
| 12 | GUGUCAUUGACGAUGAGGU UU  UU CACAGUAACUGCUACUCCA | GT GTGTCATTGACGATGAGGT CG | 3 |
| 13 | CAGGAAGAAUACUCUGCCA UU  UU GUCCUUCUUAUGAGACGGU | GA CAGGAAGAATACTCTGCCA TG | 5 |
| 14 | GUUAAGGACAAGGACUACU UU  UU CAAUUCCUGUUCCUGAUGA | GC GTTAAGGACAAGGACTACT TC | 3 |
| 15 | GACAAGGACUACUUCCCUA UU  UU CUGUUCCUGAUGAAGGGAU | AG GACAAGGACTACTTCCCTA TC | 5 |
| 16 | CAUUCCAACAACAAAUCCU UU  UU GUAAGGUUGUUGUUUAGGA | GA CATTCCAACAACAAATCCT CC | 4 |
| 17 | CUUUGAAGAAAUCAUGACA UU  UU GAAACUUCUUUAGUACUGU | TC CTTTGAAGAAATCATGACA TT | 5 |
| 18 | CUAUCAUUGUCGUCGGCAA UU  UU GAUAGUAACAGCAGCCGUU | CC CTATCATTGTCGTCGGCAA CA | 6 |

**Table S2B: List of siRNA candidates predicted in An*rasB* partial nucleotide sequence.** The partial sequences of the target gene was evaluated for presence of candidate siRNAs using the siRNA selection software. All the identified candidate siRNAs were scrutinized for silencing potency using the standard parameters. List of candidate siRNAs along with their probable mRNA target site and score of each individual siRNA were presented in the table.

| **S.No.** | **SiRNA candidate** | **Probable mRNA target site** | **Score** |
| --- | --- | --- | --- |
| 1 | GCUUCCUUCUCCAGAAUAA UU  UU CGAAGGAAGAGGUCUUAUU | GT GCTTCCTTCTCCAGAATAA CG | 8 |
| 2 | GUCCUCGUCUACAGCAUUA UU  UU CAGGAGCAGAUGUCGUAAU | TC GTCCTCGTCTACAGCATTA CG | 7 |
| 3 | GUGCUUCCUUCUCCAGAAU UU  UU CACGAAGGAAGAGGUCUUA | GC GTGCTTCCTTCTCCAGAAT AA | 6 |
| 4 | GACCAUUGAAGAUUCGUAU UU  UU CUGGUAACUUCUAAGCAUA | CC GACCATTGAAGATTCGTAT CG | 4 |
| 5 | CGAAGUUCUACAACCAAAU UU  UU GCUUCAAGAUGUUGGUUUA | AA CGAAGTTCTACAACCAAAT CA | 6 |
| 6 | GCAAGCAAGUCGUCAUUGA UU  UU CGUUCGUUCAGCAGUAACU | TC GCAAGCAAGTCGTCATTGA TC | 8 |
| 7 | CACUUCCAGUUCCCGUAAU UU  UU GUGAAGGUCAAGGGCAUUA | AC CACTTCCAGTTCCCGTAAT GC | 6 |
| 8 | CGACCAUUGAAGAUUCGUA UU  UU GCUGGUAACUUCUAAGCAU | TC CGACCATTGAAGATTCGTA TC | 6 |
| 9 | GAUCAGCAGUCAUGCAUGU UU  UU CUAGUCGUCAGUACGUACA | TT GATCAGCAGTCATGCATGT TG | 5 |
| 10 | CUCGUCUACAGCAUUACGU UU  UU GAGCAGAUGUCGUAAUGCA | TC CTCGTCTACAGCATTACGT CG | 4 |
| 11 | CGUCAUUGAUCAGCAGUCA UU  UU GCAGUAACUAGUCGUCAGU | GT CGTCATTGATCAGCATCA TG | 5 |

**Table S2C: List of siRNA candidates predicted in s*GFP* nucleotide sequence.** The partial sequences of the target gene was evaluated for presence of candidate siRNAs using the siRNA selection software. All the identified candidate siRNAs were scrutinized for silencing potency using the standard parameters. List of candidate siRNAs along with their probable mRNA target site and score of each individual siRNA were presented in the table.

| **S.No.** | **SiRNA candidate** | **Probable mRNA target site** | **Score** |
| --- | --- | --- | --- |
| 1 | UGGACGAGCUGUACAAAUA UU  UU ACCUGCUCGACAUGUUUAU | CA TGGACGAGCTGTACAAATA GG | 8 |
| 2 | GCCACAACGUCUAUAUCAU UU  UU CGGUGUUGCAGAUAUAGUA | CA GCCACAACGTCTATATCAT GG | 6 |
| 3 | ACAGCCACAACGUCUAUAU UU  UU UGUCGGUGUUGCAGAUAUA | CA ACAGCCACAACGTCTATAT CA | 7 |
| 4 | CAGCCACAACGUCUAUAUC UU  UU GUCGGUGUUGCAGAUAUAG | AA CAGCCACAACGTCTATATC AT | 4 |
| 5 | GCUGGAGUACAACUACAAC UU  UU CGACCUCAUGUUGAUGUUG | AA GCTGGAGTACAACTACAAC AG | 3 |
| 6 | AGCUGACCCUGAAGUUCAU UU  UU UCGACUGGGACUUCAAGUA | CA AGCTGACCCTGAAGTTCAT CT | 6 |
| 7 | AGCAGCACGACUUCUUCAA UU  UU UCGUCGUGCUGAAGAAGUU | GA AGCAGCACGACTTCTTCAA GT | 6 |
| 8 | AGCAGAAGAACGGCAUCAA UU  UU UCGUCUUCUUGCCGUAGUU | CA AGCAGAAGAACGGCATCAA GG | 5 |
| 9 | AGCUGGAGUACAACUACAA UU  UU UCGACCUCAUGUUGAUGUU | CA AGCTGGAGTACAACTACAA CA | 6 |
| 10 | UCAAGGUGAACUUCAAGAU UU  UU AGUUCCACUUGAAGUUCUA | CA TCAAGGTGAACTTCAAGAT CC | 6 |
| 11 | CAACAGCCACAACGUCUAU UU  UU GUUGUCGGUGUUGCAGAUA | TA CAACAGCCACAACGTCTAT AT | 6 |
| 12 | AGGUGAACUUCAAGAUCCG UU  UU UCCACUUGAAGUUCUAGGC | CA AGGTGAACTTCAAGATCCG CC | 3 |
| 13 | ACUACAACAGCCACAACGU UU  UU UGAUGUUGUCGGUGUUGCA | CA ACTACAACAGCCACAACGT CT | 4 |
| 14 | CAAGCUGGAGUACAACUAC UU  UU GUUCGACCUCAUGUUGAUG | CA CAAGCTGGAGTACAACTAC AA | 4 |
| 15 | CGAGCUGUACAAAUAGGGU UU  UU GCUCGACAUGUUUAUCCCA | GA CGAGCTGTACAAATAGGGT AC | 3 |
| 16 | ACUUCAAGAUCCGCCACAA UU  UU UGAAGUUCUAGGCGGUGUU | GA ACTTCAAGATCCGCCACAA CA | 6 |
| 17 | AGAACGGCAUCAAGGUGAA UU  UU UCUUGCCGUAGUUCCACUU | GA AGAACGGCATCAAGGTGAA CT | 8 |
| 18 | CCAUCUUCUUCAAGGACGA UU  UU GGUAGAAGAAGUUCCUGCU | CA CCATCTTCTTCAAGGACGA CG | 7 |
| 19 | CAACUACAACAGCCACAAC UU  UU GUUGAUGUUGUCGGUGUUG | TA CAACTACAACAGCCACAAC GT | 4 |
| 20 | ACGUCUAUAUCAUGGCCGA UU  UU UGCAGAUAUAGUACCGGCU | CA ACGTCTATATCATGGCCGA CA | 5 |
| 21 | GUACAACUACAACAGCCAC UU  UU CAUGUUGAUGUUGUCGGUG | GA GTACAACTACAACAGCCAC AA | 3 |
| 22 | CAACGUCUAUAUCAUGGCC UU  UU GUUGCAGAUAUAGUACCGG | CA CAACGTCTATATCATGGCC GA | 3 |
